# Spatiotemporal characterization of single-stranded DNA Intermediates after UV Irradiation: I: Post-replication gaps formed during slow growth

**DOI:** 10.1371/journal.pgen.1012109

**Published:** 2026-05-14

**Authors:** Nischal Sharma, Megan E. Cherry, Camille Henry, Elizabeth A. Wood, Andrew Robinson, Antoine van Oijen, Harshad Ghodke, Michael M. Cox

**Affiliations:** 1 Molecular Horizons and School of Chemistry and Molecular Bioscience, University of Wollongong, Wollongong, Australia; 2 Department of Biochemistry, University of Wisconsin - Madison, Madison, United States of America; Michigan State University, UNITED STATES OF AMERICA

## Abstract

When *E. coli* cells are UV irradiated, replisome encounters with some DNA lesions lead to lesion skipping and formation of ssDNA gaps. These gaps are protected by SSB and repaired through the RecFOR recombinational DNA repair pathway. However, many questions about this pathway remain unanswered. Here, we used a fluorescent SSB fusion that supports normal growth in the absence of WT SSB under most conditions to directly visualize the real-time formation and resolution of ssDNA intermediates in cells lacking factors (RecB, RecJ, RecF, and RecO), that facilitate recombinational DNA repair pathways under slow growth conditions. Upon DNA damage, SSB-bound features of various sizes increased within these cells. In WT cells, ssDNA gaps appeared and were resolved at a steady state level that persisted for hours. Formation of most ssDNA gaps was not dependent on RecB function. Large increases in ssDNA gaps were observed in cells lacking RecFORJ functions, particularly in the absence of RecO. These findings indicate that: (a) When hundreds of UV lesions are introduced into the genome, at least some lesions remain unaddressed by nucleotide excision repair (NER) for several hours under slow growth conditions. (b) Replisome encounters with DNA lesions rapidly generate ssDNA gaps. (c) A relatively small portion of the ssDNA foci appearing in WT cells may reflect breaks processed by the RecBCD system. (d) Most prominent SSB features reflect post-replication gaps repaired by RecFORJ. Lack of RecFORJ functions leads to accumulation of unresolved gaps over time. (e) RecF is not required for post-replication gap formation. Overall, the results provide direct visualization of complex UV-induced changes in DNA metabolism caused by replisome encounters with UV-generated pyrimidine dimers. Combined with a decades-long literature of related results and proposals, a unified view of how *E. coli* responds to UV irradiation can be put forward.

## Author summary

Multiple pathways exist for repairing single-stranded DNA gaps left by the replisome when it encounters UV-induced DNA lesions. In *Escherichia coli*, the RecFORJ pathway is primarily involved during the initial stages of ssDNA gap repair. Additional ssDNA gaps at DNA ends are generated when replisomes encounter template strand discontinuities and generate double strand breaks, subsequently repaired by the RecBCD system. Our live-cell fluorescence microscopy data reveal that ssDNA gap formation is a common outcome when the replisome encounters UV-induced lesions, with the RecFORJ pathway playing a crucial role in resolving the resulting post-replication gaps. Notably, the absence of RecB has a significant but not predominant effect on ssDNA gap formation. Much larger effects are seen in cells lacking any of the RecFORJ proteins. Altogether, these findings provide new insights into the distinct roles of different factors that are associated with recombinational repair. The results also allow us to put forward a scheme for how many disparate repair functions operate in a complementary fashion after a severe challenge with DNA damage.

## Introduction

Replication of chromosomal DNA is a tightly controlled, complex process that requires the assembly and action of a multi-protein complex – the replisome [1–5]. When a replication fork encounters roadblocks - such as DNA lesions, template strand breaks, or DNA bound proteins – the many outcomes include replisome stalling and collapse or lesion bypass [6–13]. In bacteria, replication forks may stall as often as once per cell generation during normal growth conditions [7,8,14] and bypass lesions to form post-replication gaps multiple times [7,15]. For cells to survive, it is imperative that they are able to recognize, respond to, and resolve these conflicts in an efficient, preferably non-mutagenic, fashion such that genomic integrity and cell viability are maintained.

A role for post-replication gaps in the repair of certain types of DNA damage was first hypothesized by Rupp and Howard-Flanders [16]. Upon encountering bulky DNA lesions, especially on the lagging strand template, replisomes may bypass the damaged site by skipping ahead [7,15]. Lesion skipping allows for uninterrupted replication [17]. However, this leaves behind a lesion-containing post-replication ssDNA gap that must be resolved. First, the gap intermediate is coated by ssDNA binding protein (SSB) [18]. Once coated, gaps can then be resolved in one of three ways – RecA dependent homologous recombination (the primary path) [7,19–22], error prone translesion DNA synthesis [7,19–22], or RecA-independent template-switching [7,23–25].

When cells are subjected to UV irradiation, lesions, mainly cyclobutane dimers and (6–4) photoproducts, are introduced into the chromosomal DNA at approximately 0.7 lesions per 100 kbp per J/m^2^ exposure [16,26,27]. Experimentally, these are the classic triggers for post-replication gap formation [7]. At the 5–50 J/m^2^ doses commonly used in past studies, this would amount to about 160–1600 lesions per *E. coli* genome, or one every 3,000–30,000 bp. Active replisomes would begin encountering these lesions within seconds. Beginning 5–10 min after irradiation, replication halts for a period of up to 20–80 minutes prior to resumption [13,28–32]. Recovery of replication elongation requires RecA [31,33,34], the RecFOR functions [29,30,35–37], RecJ [38], the nucleotide excision repair system [30,35,39], and PriA [40,41]. The molecular cause of the halt in replication has not been elucidated in detail but presumably represents replisome encounters with damage. NER will produce transient breaks in the strand where a pyrimidine dimer is removed; a replisome encounter with that strand discontinuity should result in replisome collapse. Lesion-skipping occurs, but its contribution to the overall course of lesion amelioration relative to other processes has not been clear.

Genomic ssDNA increases substantially after UV irradiation [42]. Nucleotide excision repair (NER) would begin to address lesions throughout the chromosome [13,43–49], but at varying rates in different genome locales [44,45,50]. Via transcription-coupled repair, the NER system is directed first to lesions the transcribed strand of actively transcribed genes [46,47]. Lesions in other locales are repaired more slowly [46–49]. Replisomes encountering template discontinuities at lesions undergoing NER would trigger double strand breaks. The various ssDNA intermediates generated as replisome encounters generate breaks and gaps lead to induction of the SOS response [51,52]. This general picture of the effects of UV irradiation, established over multiple decades of research, is generally based on indirect methods. No methods have been available for direct observation of ssDNA generation and resolution. Some replisome encounters with lesions clearly result in a halt in replication even as some lesions may be bypassed.

In bacteria, recombinational repair is mediated by RecA protein [7,53–56]. First, RecA, bound to ATP, with the aid of recombination mediator proteins (RMPs), displaces SSB to form activated nucleoprotein filaments on ssDNA gaps [7,52]. Through homology search, the RecA filaments promote strand exchange and branch migration to create a joint molecule linking the daughter DNA molecules behind the fork [7,10,54,57]. Additionally, active RecA filaments exhibit a co-protease activity by facilitating auto-cleavage of LexA protein, a repressor of the SOS regulon that induces the DNA damage signaling cascade termed the SOS response [58–60]. The SOS response is a global response to increased levels of DNA damage that allows bacterial cells to attempt DNA repair first via error-free homologous recombination. Then, if necessary, damaged cells resort to error-prone translesion synthesis (TLS) [60–63].

In *E. coli*, displacement of SSB from ssDNA gaps formed by lesion-skipping is facilitated by the coordinated action of the RMPs RecJ, RecF, RecO, and RecR via the RecFOR pathway [64–66]. Recent studies indicate that the RecJ nuclease expands the ssDNA gaps during the early phases of post-replication gap repair [15,20,22,67]. Despite many studies linking the RecFOR activities, to date no stable RecFOR complex has been observed. Both *in vivo* and *in vitro* studies have shown that the RecF and RecO proteins separately complex with RecR protein to form the RecFR and RecOR complexes [68–76]. These two complexes occupy distinct spatiotemporal locations within the bacterial cell [64,72,77]. Recently, a direct interaction between RecF and the DnaN β-clamp was identified [78], This interaction may facilitate RecF’s targeting of lesion-containing post-replication gaps, with potential impact in the ssDNA gap formation [79]. Therefore, the RecFR complex exhibits significant co-localization with the replisome, acting as a targeting function to facilitate identification of lesion-containing gaps requiring repair [7,78]. The RecOR complexes promote disassembly of SSB from ssDNA gaps to enable the loading of RecA [64,65,73–75,80]. At some point, RecFR must give way to RecOR, possibly via a hand-off of RecR from RecF to RecO. RecA is then loaded into the gap by RecOR complexes [68,69,72,73,81]. Subsequent steps in recombination are likely facilitated by the alignment of sister chromatids for a period after replication, requiring RecN [82–84].

Unlike dsDNA, ssDNA is less stable and highly flexible [85]. After UV irradiation, single strand gaps of significant size are likely to be created not only via lesion-skipping but also via the action of RecBCD in double strand break repair. The double strand breaks would reflect replisome encounters with template discontinuities created by ongoing NER instead of an intact UV lesion [86]. Double-stranded DNA break repair (DSBR), mediated by the RecA-dependent RecBCD pathway [87–90], creates single stranded gaps as the RecBCD nuclease/helicase degrades the exposed ends of dsDNA breaks to ssDNA intermediates. In the absence of RecBCD, the RecFOR pathway may participate in DSBR beginning with the coordinated action of the RecJ and RecQ proteins [91]. This is a secondary role for RecJ as its primary function appears to lie in ssDNA gap repair via the RecFORJ pathway [20,92], a function that does not rely on RecQ [15].

Given the complicated interplay of so many pathways, the study of DNA gaps, post-replication gaps in particular, has become an increasingly important part of understanding bacterial genome maintenance and metabolism. Despite a revolution in cell-imaging technology in recent decades, there has been a lack of tools to directly observe post-replication gaps in live cells. Fundamental questions that remain unanswered include: (a) how often are lesions bypassed relative to how often they halt replication, (b) how do various recombination factors orchestrate ssDNA gap repair during recombinational repair within cells following UV DNA damage, (c) to what extent do various recombinational repair processes contribute to DNA metabolism under particular DNA damage circumstances, (d) what is the fate of DNA lesions when repair is compromised, (e) how long do various repair processes take, and (f) how does the generation of post-replication gaps fit into the overall response to UV damage?

In this study, we begin to address these questions using a novel fusion of SSB which has a fluorescent protein, mTurquoise2 (mTur2), incorporated in the IDL (intrinsically disordered linker) domain (between amino acids F148 and S149) to directly visualize ssDNA intermediates inside live cells after UV exposure [93]. Developed by the Keck laboratory, these novel SSB fluorescent fusions can replace the wild-type copy of the *ssb* gene on the chromosome with minimal effects on cell growth and DNA damage sensitivity [93].

What sorts of gaps might we see with these methods? The gaps created during double strand break repair are *created* by RecBCD, so gaps generated by this process should decline in a *recB* mutant. Post-replication gaps created by lesion-skipping are *resolved* by the RecFORJ pathway, so gaps generated by lesion-skipping should increase in a mutant lacking a *recFORJ* function. Short gaps produced by NER are unlikely to be large enough to bind SSB. A low level of gaps generated by mismatch repair may also be present but this level should be minimally affected by UV irradiation.

We used live-cell fluorescence microscopy to image a series of mutant strains of *E. coli* containing the SSB-mTur2 fusion and that were deficient in either RMPs (RecJ, RecF, and RecO) or the DSBR protein (RecB). Imaging revealed that upon exposure to UV, wild type cells exhibited numerous smaller and larger ssDNA-bound SSB features that were both atypical of those observed for replication forks under normal growth conditions and relatively rare in the absence of DNA damage under our slow growth conditions. Thus, they likely reflect ssDNA products generated as a result of lesion-skipping or DSBs. The increase in UV-induced SSB features persisted for almost 3 hours within wild-type cells. Under our slow growth conditions here, this corresponds to about one cell cycle equivalent. The effects of UV changed in the mutant cells. Consistent with previous observations, RecB-deficient cells were unable to filament after exposure to UV [79,94]. Cells lacking RecB exhibited somewhat fewer DNA-bound SSB features after UV than in wild type cells, reflecting a significant but not dominant role for RecB in the generation of SSB foci in post-UV DNA metabolism. In contrast, the elimination of both RecF and RecO led to significant deficits in gap resolution as indicated by large increases in the size and numbers of SSB foci, highlighting the importance of RecF and RecO in resolving observed ssDNA intermediates. These large increases also persisted for multiple hours. Overall, our study provides direct observation of the complex dynamics of SSB-bound ssDNA gaps formed within bacterial cells in the presence or absence of recombination proteins upon exposure to UV.

## Results

### Recombination-deficient cells expressing SSB fluorescent fusions are sensitive to UV

**Fig 1:**
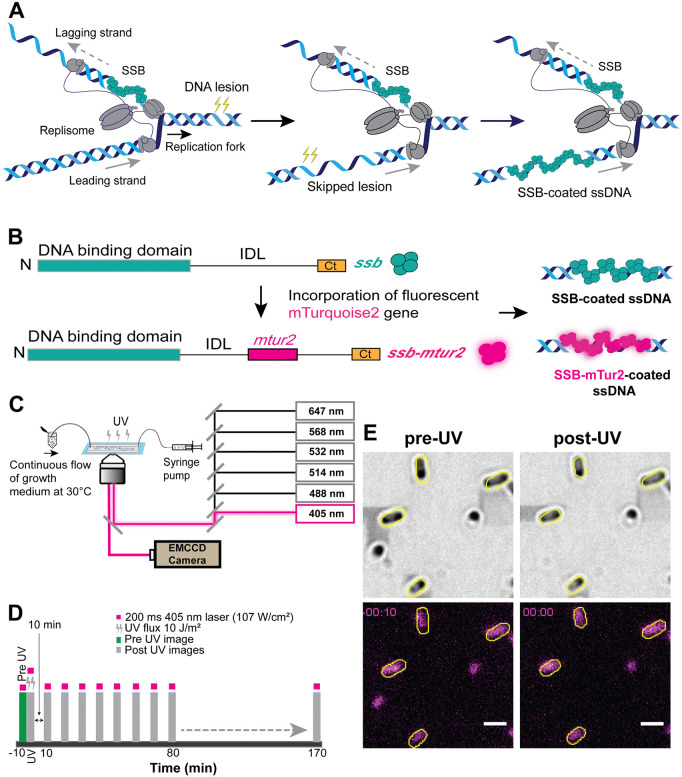
Experimental design for live-cell imaging of fluorescent *E. coli* constructs. (**A)** Schematic representation of replisome skipping DNA lesion during replication forming SSB-coated ssDNA gaps. **(B)** Schematic of wild-type SSB and SSB internal fusions with DNA binding domain (grey), C-terminal domain (Ct, yellow), IDL and fluorescent protein mTurquoise2 (mTur2, magenta). The fluorescent SSB probe (*ssb-mTur2*) was used in this study to visualize the DNA gaps after DNA damage **(C)** Experimental setup for imaging the cells using an inverted TIRF microscope. Immobilized cells continuously supplemented with M9 minimal medium including 20 mM glucose were illuminated with a 405 nm laser (power density: 107 W/cm^2^) in highly inclined laminated optical sheet (HILO) mode. **(D)** Time-lapse interval imaging scheme. 256 nm UV light at a flux of 10 J/m2 was delivered in situ at time = 0. The pre-UV image was recorded prior to UV exposure, and the post-UV images were recorded at 10-minutes intervals for 170 minutes. **(E)** Brightfield (top) and 405 (bottom) images of WT cells before UV exposure (left images), immediately after UV exposure (right images). Scale bar represents 2 µm.

**Fig 2:**
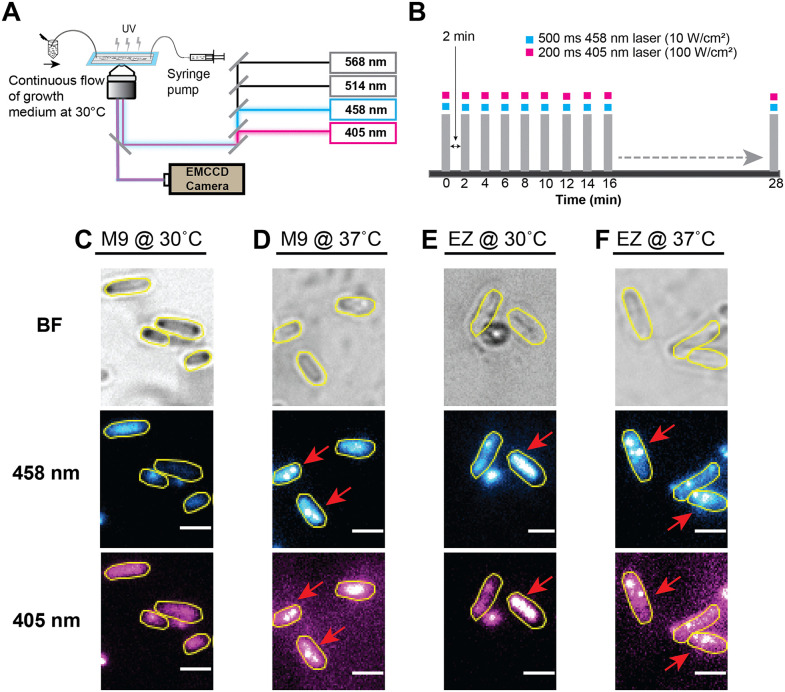
Experimental setup for double imaging of WT-*ssb-mTur2* strains (EAW1169). **(A)** Immobilized cells were imaged with an inverted wide-field microscope using 405 nm and 458 nm lasers. **(B)** Interval imaging scheme to examine the formation of DNA-bound SSB-mTur2 foci inside WT cells supplemented M9 minimal and EZ-rich medium at 37 °C. Time-lapse imaging of these immobilized cells (grown in M9 minimal and EZ-rich media at 37 °C was performed using 405 nm and 458 nm lasers. Data acquisition settings for recording the time-lapse images with a 405 nm laser were unchanged (100 W/cm^2^, 200 ms), whereas data acquisition using a 458 nm laser was kept similar to the settings used by Cherry et al., 2023 (10 W/cm^2^, 500 ms). All cells were grown in M9 minimal and EZ-rich media, at either 30 °C or 37 °C were recorded without UV exposure. Cells grown in EZ images were recorded every 2-minute interval for 30 minutes. **(C)** Double imaging of WT cells supplemented with M9 minimal medium at 30 °C, **(D)** M9 minimal at 37°C, and **(E)** EZ-rich medium at 30°C and, **(F)** EZ-rich medium at 37°C. The red arrow shows the formation of at least one focus within the cells. Scale bar represents 2 µm.

**Fig 3:**
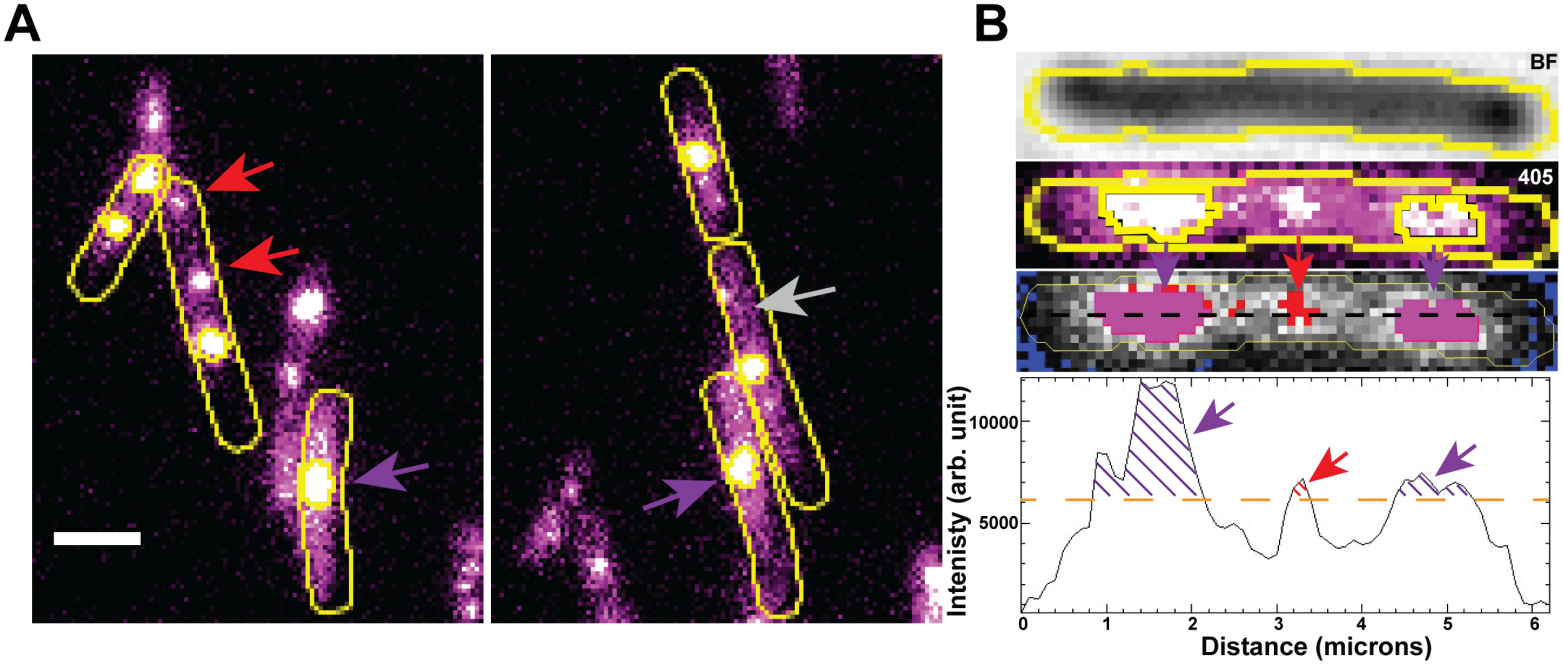
Characterization of fluorescent SSB features inside the cell. **(A)** Bacterial cells illuminated with a 405 nm laser after UV exposure. The grey arrow indicates diffused, unbound proteins in the cytosol. The red arrow points to diffraction-limited foci, and the purple arrow shows large clusters of bound SSB foci. **(B)** Top: Brightfield image of the cell. Middle: 405 nm laser image of the cell. Bottom: line section of the cell (black dashed line) and the corresponding fluorescent intensity plot (bottom graph). The red arrow highlights diffraction-limited foci with an area of 16 pixels² (0.16 µm²). Purple arrows indicate large clusters of bound SSB foci with an area >16 pixels² (>0.16 µm²). The grey arrow points to diffused unbound SSB proteins in the cytosol (background), which were filtered out by applying a threshold intensity of 6100 arbitrary units (orange dashed line) to all images. Scale bar represents 2 µm.

The present study begins with a detailed method validation, with results described in Figs 1-3 and several supporting figures. In brief, the SSB-mTur2 fluorescent fusion protein has little effect under normal growth conditions. Small effects are noted with UV exposures higher than those used in this study, increasing when certain repair factors (especially RecB) are absent.

Previous studies reported that the SSB-mTur2 fluorescent fusion protein used in this study supported cell survival comparable to wild-type (WT) levels in response to low UV dose (10J/m^2^) [79,93]. To determine if the chromosomal SSB fusions alter the DNA repair activities of recombination-deficient strains (Δ*recB ssb-mTur2*, Δ*recJ ssb-mTur2*, Δ*recF ssb-mTur2*, Δ*recO ssb-mTur2* and Δ*recF* Δ*recO ssb-mTur2*), a UV-sensitivity assay at 5 J/m^2^, 10 J/m^2^, and 15 J/m^2^ was performed. UV exposure to 5–15 J/m^2^ should introduce between 160–500 pyrimidine dimers per genome, with perhaps a quarter of those being (6–4) photoproducts [16,26,27]. Recombination-deficient strains without SSB fusions (Δ*recB*, Δ*recJ* and Δ*recF*Δ*recO*) were used as controls (See the list of *E. coli* strains used in this study in S1 Table). None of the SSB fusion-expressing strains, except the Δ*recB ssb-mTur2* strains, showed any differences in UV sensitivity compared to WT at a low UV dose (5 J/m^2^) or when no UV was applied (S1A and S1B Fig). At 10 J/m^2^ (the UV dose that we applied in the imaging experiment), WT-*ssb-mTur2* and Δ*recJ ssb-mTur2* strains displayed a similar UV-survival profile compared to WT, whereas recombination mediator protein-deficient cells with chromosomal SSB fusion (Δ*recF ssb-mTur2*, Δ*recO ssb-mTur2* and Δ*recF* Δ*recO ssb-mTur2*) were more sensitive to UV by one order of magnitude. The effect on *ΔrecB ssb-mTur2* cells was greater. Further, an increase in UV dose to 15 J/m^2^ led to increased sensitivity within the Δ*recB ssb-mTur2,* Δ*recF ssb-mTur2*, Δ*recO ssb-mTur2* and Δ*recF* Δ*recO ssb-mTur2* strains, while WT-*ssb-mTur2* and Δ*recJ ssb-mTur2* strains displayed an equal degree of sensitivity as compared to WT cells. Altogether, the results suggest that the fluorescent SSB fusion-expressing cells, except Δ*recB ssb-mTur2*, maintain near-wild type activity in M9 minimal medium under the UV conditions that we used in the imaging experiment. However, growth-curve measurement based on optical density showed that all cells with SSB-mTur2 background lacking recombination proteins exhibited a longer lag in growth compared to WT (without fluorescent *ssb-mTur2* gene) cells and cells with a chromosomally incorporated *ssb-mTur2* gene (EAW1169), in the absence of UV (S1C Fig). Notably, we found a prolonged lag in cell growth after UV exposure (10 J/m²), with Δ*recB ssb-mTur2* strains demonstrating a significantly extended lag phase compared to all other strains (S1D Fig). Because of this slower growth, the recombination-deficient cells with SSB-mTur2 background were incubated for an additional 3–4 hours compared to WT-*ssb-mTur2* strains to maintain the early-mid exponential growth phase before imaging the cells. We note that doubling times for most strains were similar to WT cells (~3 hours) once exponential growth was achieved. However, the results obtained for the Δ*recB ssb-mTur2* strains should be viewed with the realization that many, if not most of the cells will eventually die.

### Experimental set up for live-cell imaging of *Escherichia coli*

UV light generates DNA intramolecular crosslinks (6–4 photoproducts and thymine dimers) that impede DNA replication leading to the accumulation of single-stranded DNA gaps inside the cell [39,95,96] (Fig 1A). To visualize the formation of SSB-coated ssDNA gaps, we performed time-lapse imaging of individual *E. coli* constructs, encoding SSB proteins which has a fluorescent mTurquoise2 integrated in the IDL domain between amino acids F148 and S149 (Fig 1B), immobilized in flow cells (for imaging details, see the materials and methods section) growing in the presence of fresh, aerated M9 minimal medium at 30°C (Fig 1C). We used M9 minimal medium, including 20 mM glucose, as the preferred growth medium for cells for two reasons: 1) bacterial cells grown in M9 were able to adhere to the surface of functionalized coverslips for a longer duration than those grown in EZ-rich medium and, 2) M9-grown bacteria displayed many fewer fluorescent SSB features than EZ-grown bacteria [97]. This difference is attributed to the slower growth rate in M9 medium, resulting in fewer concurrent DNA replication events, which enables clearer characterization and quantification of SSB-coated ssDNA. If a replication cycle requires an hour or less, and the doubling time is 3 hours, only a subset of cells, perhaps a third of them, should have active replication forks [98]. The lagging strand gaps produced by active replication forks may also be less visible under these conditions. Overall, the number of foci observed per cell prior to UV irradiation was much reduced relative to the cells grown at 37 °C in the previous work of Dubiel et al [93].

The time-lapsed imaging of cells was performed as follows: first, cells from 35-45 distinct fields of view within a single flow channel were imaged using 405 nm bright-field illumination. Next, a pulse of UV (10 J/m^2^) was delivered *in situ* at time = 0 min, and finally, the same fields of view were further imaged in both channels (Brightfield and 405 nm) at intervals of 10 min for 170 min (Fig 1D). The longer timescale imaging interval was used in this study for capturing long-lived SSB binding events and further decreasing the risk of photodamaging of cells from exposure to high laser power (107 W/cm^2^).

### WT-*ssb-mTur2* cells displayed fewer SSB foci prior to DNA damage in the minimal medium

As described below, we have chosen growth conditions and visualization methods that minimize foci detection from active replication forks before UV irradiation. This allows us to focus on foci that reflect the effects of replisome collisions with new pyrimidine dimers. Most detectable SSB foci are thus most likely ascribed to the formation of post-replication gaps or to the processing of DNA ends at double strand breaks by RecBCD.

First, we imaged cells expressing chromosomal *ssb-mTur2* in a wild-type background (EAW1169) grown in M9 minimal media. Prior to DNA damage (and immediately after UV exposure, at t = 0 min), very few cells formed DNA-bound SSB foci detectable under the conditions and methods employed here (Fig 1E). We found that only 2% of WT- *ssb-mTur2* strains displayed SSB foci per cell under pre-UV conditions (S2A Fig). This finding differed from previous observations by Dubiel et al. [93] and Cherry et al. [79], where they initially noted the development of at least two SSB foci within cells reflecting probable replication forks, under a different set of growth and observation conditions using the same SSB-mTur2 IDL fusion [79,93]. A relatively low number of pre-UV foci (replisome-associated) observed inside cells under our experimental conditions could likely attributed to three different factors: 1) greater cellular autofluorescence compared to the SSB-mTur2 IDL fusion fluorescence, 2) cell images acquired with a 405 nm laser could result in pre-UV foci below the threshold intensity value (an intensity value assigned to capture fluorescent SSB features inside the cell by filtering out the fluorescent cytosolic SSB signal), and 3) growth conditions. We carried out a series of experiments to determine in more detail why and how the results in the present study differed from previous results in the studies by Dubiel et al [93] and Cherry et al [79].

Increased cellular autofluorescence due to the natural background emission of light from cellular components can interfere with the detection of SSB-mTur2 IDL fusion protein fluorescence. This interference could be further complicated by the differences in the application of imaging tools, laser wavelengths, or cellular growth conditions, all of which can influence the intensity and spectral characteristics of both the autofluorescence and the fluorescent protein signal. The previous study by Dubiel et al. utilized an epifluorescence microscope [93] to detect fluorescent SSB foci, while Cherry et al. employed a custom-built wide-field fluorescence microscope with a 458 nm laser [79] to detect the fluorescent SSB features within WT-*ssb-mTur2* cells. In contrast, our study used a custom-built inverted fluorescence microscope equipped with a 405 nm laser to illuminate WT-*ssb-mTur2* cells for detecting the SSB foci. The use of different imaging equipment or a different laser under our imaging set up may have resulted in the formation of lower-intensity pre-UV foci at replication forks that fell below the detection threshold.

To assess if the reduced pre-UV foci observed in WT-*ssb-mTur2* strains under the imaging conditions used in this study were due to increased cellular autofluorescence, (potentially caused by the use of different laser excitation wavelengths or variations in nutrient media), we performed a control experiment by double imaging single-color WT-*ssb-mTur2* cells (EAW1169) under four different conditions using a wide-field fluorescence microscope equipped with both 458 nm and 405 nm lasers (see the imaging details in the methods section “Single-molecule live cell imaging”) (Fig 2A). The purpose of double imaging WT-*ssb-mTur2* cells using 405 nm and 458 nm lasers is to determine whether the fluorescent SSB foci are visible in one or both channels, addressing concerns about potential autofluorescence artifacts. First, we imaged WT-*ssb-mTur2* cells maintained in M9 minimal medium at 30°C (under conditions similar to those used for imaging all cells in this study) to verify whether these cells truly display few SSB foci before UV exposure. Second, WT-*ssb-mTur2* strains grown in M9 minimal medium at 37°C were imaged to examine whether SSB foci develop under minimal growth conditions, as observed by Dubiel et al. [93]. Third, these strains grown in EZ-rich medium at 37°C were imaged to assess whether more SSB foci develop under nutrient-rich conditions, as noted by Cherry et al. [79]. Lastly, cells maintained in EZ-rich medium at 30°C were imaged to determine whether the observed pre-UV foci are influenced by nutrient availability and temperature variations.

The double time-lapse imaging of bacterial cells grown in M9 minimal or EZ-rich media at 30 °C was recorded at every 10 min interval for a period of 170 min without exposing the cells to UV (Fig 1D). Cells cultured in M9 minimal or EZ-rich media at 37 °C were imaged at 2 min intervals for 28 min without DNA damage (Fig 2B). The imaging setup using a 458 nm laser was kept comparable to the conditions used by Cherry et al. [79], while data acquisition using a 405 nm laser was unchanged. We found that the temperature and growth medium, rather than the excitation with different lasers, influence the development of DNA-bound SSB foci within cells. For all four imaging conditions, only WT-*ssb-mTur2* cells (N > 50) from the first frame (at the time point 0 sec) were selected, and the percentage of cells displaying the pre-UV SSB foci was quantified.

Double time-lapse imaging revealed that approximately 8% of WT-*ssb-mTur2* cells grown in M9 minimal medium at 30°C exhibited pre-UV foci (Figs 2C and S3A), consistent with our observations in this study (S2A Fig). However, minor variations in the fraction of pre-UV foci observed under identical growth conditions likely reflect differences in sample size, imaging parameters, and cell-stage synchronization inherent to live-cell microfluidic experiments, and do not alter the overall conclusion that only a small subset of wild-type cells display SSB foci prior to UV exposure. In contrast, cells grown in M9 minimal medium at 37°C showed a significantly higher occurrence of DNA-bound SSB foci, with nearly 98% of the population exhibiting one or more distinct foci (Figs 2D and S3A) Notably, 100% of cells grown in EZ-rich medium at 37°C displayed these DNA-bound SSB foci (Figs 2F and S3A). These findings align with the earlier studies of Dubiel et al. [93] and Cherry et al. [79], suggesting that the growth conditions influence the formation of SSB foci. Additionally, we observed that 72% of cells cultured in EZ-rich medium at 30°C exhibited similar pre-UV SSB features (Figs 2E and S3A), indicating that the proportion of cells naturally displaying SSB features at optimal temperatures is reduced at sub-optimal temperatures. Control imaging experiments further confirmed no significant differences in the detection of DNA-bound SSB foci between cells imaged using different lasers (Figs 2C-2F and S3A). This consistency in the detection of fluorescent SSB features within cells indicates that the choice of laser did not influence the detection or quantification of DNA-bound SSB foci, thus validating the robustness and reliability of our experimental approach.

Ferullo and Lovett [99] carried out flow cytometry studies that suggest that under our conditions (M9 + glucose and 30 °C), less than 1/3 of all cells should be undergoing replication based on cellular DNA content.

Based on these results, it is clear that a nutrient-rich environment and optimal temperature are crucial for the facile detection of SSB foci with SSB-mTur2, while these features minimally appear under nutrient-limited conditions or at suboptimal temperatures. Thus, we suggest that under the imaging conditions employed in this study, where most cells are not actively replicating, the normal ssDNA gaps at the replication fork are not present in most cells. The fraction of cells with visible SSB foci is even smaller than might be expected due to cell doubling times. Thus, SSB foci that represent replication forks may underestimate the number of active forks present. Having established that cells display fewer SSB foci before DNA damage in minimal medium at 30°C, we further examined the frequency of fluorescent SSB features displayed within SSB-IDL fusion-expressing cells after DNA damage.

### Percentage of WT-*ssb-mTur2* cells exhibiting SSB features of various sizes increases following UV exposure

After irradiating with a low UV dose (10J/m^2^), cell images were recorded at every 10 min time interval for 170 min to visualize the SSB-mTur2 signals bound to the ssDNA. Following UV irradiation, cell length gradually increased, in contrast to no change observed in non-irradiated cells (S3B Fig). At the end of imaging, WT-*ssb-mTur2* cells exhibited a two-fold increase in cell length compared to pre-UV levels (S3B Fig).

Due to factors described above, cells exhibited a very low background of SSB features prior to UV irradiation. The proportion of WT-*ssb-mTur2* cells showing fluorescent SSB foci increased from 2% prior to DNA damage to 4% immediately following UV exposure (S2A Fig). SSB foci then soon accumulated. The fluorescent SSB-IDL fusion-expressing cells exhibited well-defined SSB features of various sizes upon exposure to UV as observed by Cherry et al. [79]. Mainly, three different types of fluorescent SSB features were observed (Fig 3A): 1) large SSB clusters (indicated by purple arrow), 2) diffraction-limited foci (indicated by red arrow) and 3) a diffuse signal in the cytosol (indicated by gray arrow). The diffuse signal was not further quantified. The clusters and foci formed within the bacterial cells were classified based on size, following a similar approach as adopted by Cherry et al. [79] (See characterization of fluorescent SSB signals inside cell in the Methods section). Here, we refer to the large “clusters” of SSB foci (indicated by the purple arrow in Fig 3a and 3b) as SSB features with an area of greater than 16 pixel^2^ or 0.16 micron^2^ (above the threshold intensity of 0–6100 arbitrary units as indicated by the orange dashed line) above the cytosolic background, while the diffraction-limited foci (indicated by the red arrow in Fig 3a and 3b) as SSB features with an area of 16 pixel^2^ (4 x 4 pixel^2^ or 0.16 micron^2^) or less (Fig 3B). The true identity of these SSB foci and clusters cannot be assigned based on analysis of WT*-ssb-mTur2* cells alone. However, their formation predominantly after UV suggests the formation of SSB-coated ssDNA repair intermediates formed as active replisomes encounter UV lesions. We quantified the frequency of both SSB foci and clusters displayed by the bacterial cells (including WT and recombination protein-deficient strains) following UV exposure (10 J/m^2^) at every 10-min interval for 170 min. In conjunction with this analysis, we used time-lapsed imaging to characterize cell morphology (by measuring cell length), SSB density within SSB features (by measuring the mean pixel intensities of both SSB foci and clusters), and cluster size formed inside the cells following DNA damage over time.

### Elimination of *recB* reduced the cell’s ability to form filaments and DNA-bound SSB features after UV exposure

**Fig 4:**
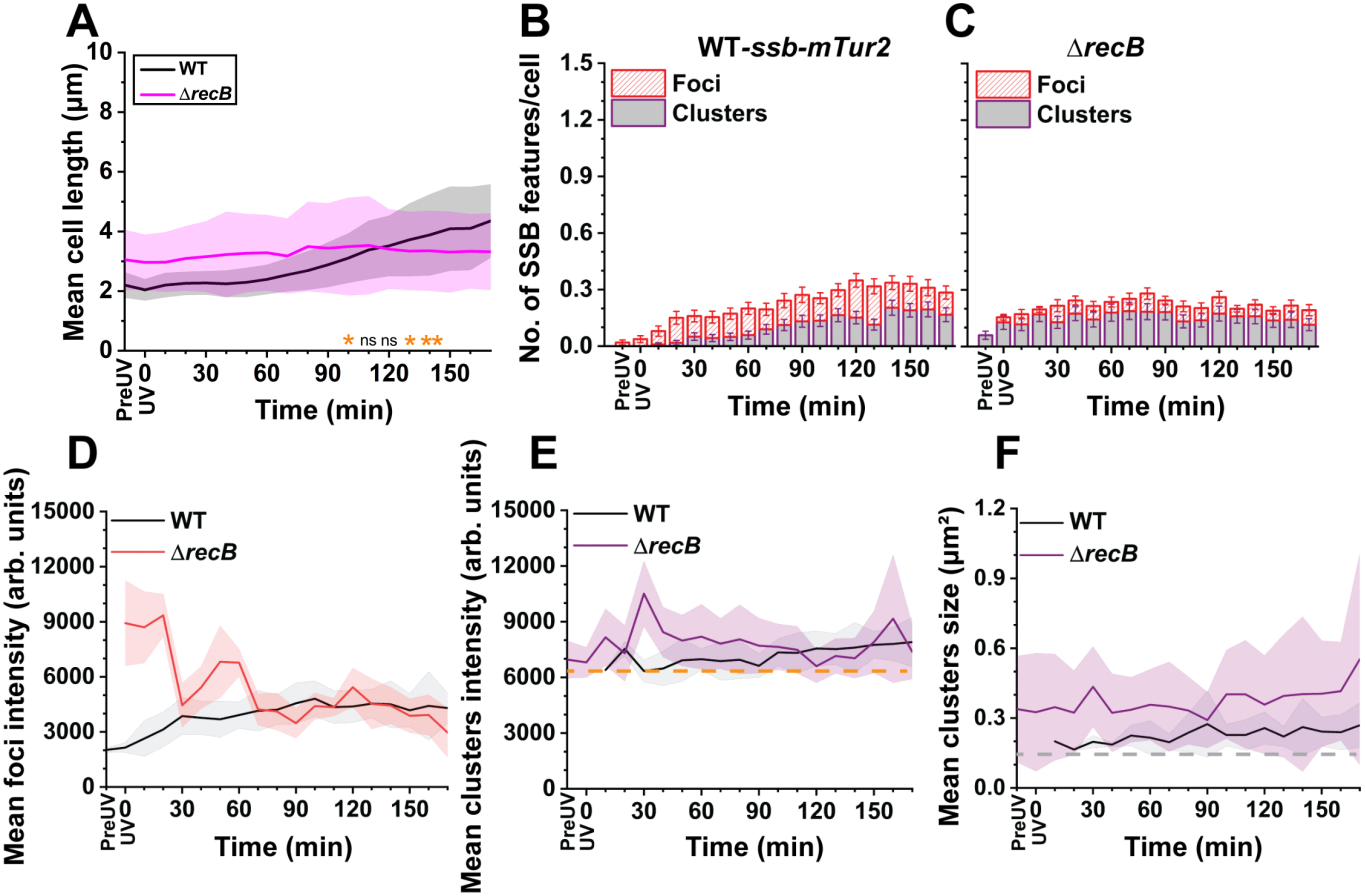
Quantification of cell length, DNA-bound SSB features (foci and clusters), foci intensity, cluster intensity, and cluster size over time (170 minutes) in response to UV exposure in WT-*ssb-mTur2* and *ssb-mTur2* Δ*recB* cells (N > 100 cells). **(A)** Cellular length of WT-*ssb-mTur2* and *ssb-mTur2* Δ*recB* cells measured over time. Shaded areas represent the standard deviation (SD) of cell length, reflecting variability within the cell population at the indicated time points. Significance is denoted (in the Table C in S1 Text) as follows: ns = not significant; * *p* < 0.05; ** *p* < 0.01. Time points with no ‘*’ or ‘**’ or “ns” indicate *p* < 0.001 in the figure (***); exact *p*-values and number of cells are reported in Table C in S1 Text. **(B)** Quantification of ssDNA-bound SSB features (foci and clusters) in WT-*ssb-mTur2* cells and, **(C)** *ssb-mTur2* Δ*recB* cells over time. The red-striped box indicates SSB foci, and the purple box indicates SSB clusters. Error bars represent the SEM for the number of foci or clusters formed within cells at theindicated time point. **(D)** Mean intensities of SSB foci in WT-*ssb-mTur2* and *ssb-mTur2* Δ*recB* cells over time. **(E)** Mean intensities of SSB clusters in WT-*ssb-mTur2* and *ssb-mTur2* Δ*recB* cells over time. The orange dashed line represents the threshold intensity value (0–6100 arbitrary units) used to filter out cytosolic SSB signals for capturing SSB clusters. **(F)** Mean SSB cluster sizes in WT-*ssb-mTur2* and *ssb-mTur2* Δ*recB* cells over time. The grey dashed line marks the minimum cluster area (>0.16 µm² or 16 pixels²). The shaded areas in **(D)**, **(E)**, and **(F)** represent the SD, highlighting variations in intensity and size within the cell populations at the indicated time points.

Following UV exposure in WT-*ssb-mTur2* and Δ*recB ssb-mTur2* (EAW1219 or RecB-deficient) cells, we first observed different effects on cellular morphology marked by cell length. We found that WT-*ssb-mTur2* cells exhibited a 2-fold increase in cell length compared to pre-UV levels. In contrast, although RecB-deficient cells displayed greater cell length prior to UV exposure, they did not form extensive filaments after DNA damage, as observed by Cherry et al. [79] (Fig 4A and Movies A and B in S1 Movie). Moreover, imaging non UV-irradiated WT-*ssb-mTur2* cells (under similar imaging conditions for 170 min) revealed that the cells did not form filaments over the 170-min observation period (S3B Fig and Movie A in S2 Movie).

Next, beginning with wild type cells, we measured the frequency of SSB features (both foci and clusters) developed within these cells before and after DNA damage. The time-lapsed images showed that immediately after UV exposure, the frequency of SSB foci and large SSB clusters increased for the first 30 min within the WT-*ssb-mTur2* cells, prior to a steady state that largely persisted until the end of the 170-minute period (Fig 4B). The levels of SSB foci and clusters appeared in approximately a third of the cells at their maximum occurrence. SSB features, particularly the smaller ones, began to decline at later times.

We tentatively interpret the results to reflect replisome encounters with UV lesions in that subset of cells with active replisomes. If a small minority of the cells have active replisomes at the time of irradiation, most of the active replisomes are encountering lesions fairly rapidly. SSB features increase during the first 20–30 min as replication forks initiate in additional cells. The ssDNA features are being constantly resolved, albeit somewhat slowly. A kind of steady state is achieved in which encounters continue to occur but are getting slowly resolved. As encounters continue throughout the 170 min of the experiment, at least some UV lesions must persist for at least the nearly 3 hours of observation time (approximately one cell cycle equivalent). Thus, nucleotide excision repair of the hundreds of lesions introduced into each genome by the UV is insufficient to clear them completely over this time course, at least under these slow growth conditions. Direct measurements in vivo indicate efficient removal of pyrimidine dimers within 30–40 min after UV irradiation [13,43]. These measurements were generally carried out under rapid growth conditions using richer media and/or higher temperatures than used here. Slow growth conditions may impede nucleotide excision repair or some lesions may be relatively inaccessible. We note that recombinational DNA repair of post-replication gaps does not actually eliminate the lesion within them, but instead simply supplies a complementary strand to fill in the gap. Thus, the same lesions may be encountered by replisomes in multiple cell generations.

In the RecBCD pathway for double strand break repair, RecB is involved in generating the ssDNA that would be detected with SSB-mTur2. Thus, if RecB is not present, the level of observed SSB foci should decline. In RecB-deficient cells, the frequency of both types of SSB features rose almost immediately after UV irradiation and remained relatively constant for the remainder of the imaging timecourse (Fig 4C). We found that a somewhat higher level of the large SSB clusters were already present before UV exposure within RecB-deficient cells, whereas smaller SSB foci only developed after DNA damage. Over the entire time course, somewhat greater numbers of SSB features formed in the WT cells relative to the cells lacking RecB, primarily in the formation of foci (S2A and S2B Fig). However, the difference was small, perhaps 30% or less. The reduced number of RecB-independent SSB features in cells lacking *recB* could be due to their slower growth rate (S1C and S1D Fig), which likely results in less frequent DNA replication and, consequently, fewer encounters between the replication machinery and DNA lesions. The reduction in SSB foci and the evident growth effects reflects a significant role for RecBCD in post-UV DNA metabolism. However, there is still a substantial level of SSB foci in these cells that are not generated by RecBCD. We reiterate that RecBCD is needed to generate the ssDNA intermediates that occur in double strand break repair, whereas the ssDNA intermediates formed by lesion-skipping do not require RecFORJ but instead are later repaired by RecFORJ. In the absence of RecB to form ssDNA intermediates at double strand breaks, many of the new foci formed in *recB* cells after UV irradiation are likely to reflect the formation of post-replication gaps.

SSB foci and clusters in WT cells accumulate progressively following UV exposure, reflecting the recruitment of SSB to transient ssDNA regions generated during DNA repair and replication-associated stress. In contrast, although RecB-deficient cells display somewhat fewer SSB features overall, likely due to their slower growth and reduced replication activity, a higher (albeit still small) proportion of the cells exhibit SSB clusters prior to UV treatment. Under normal growth, this likely reflects the persistence of unresolved ssDNA intermediates arising from impaired processing of stalled or collapsed replication forks in the absence of RecBCD activity. As a result, UV exposure leads to a smaller relative increase in SSB features in the RecB mutant, giving rise to the apparent predominance of clustered SSB signals under identical damage conditions.

We quantified the mean intensities of SSB foci (monitored by measuring the mean pixel intensities of SSB foci) within the cells lacking RecB. We observed that the mean SSB foci intensity gradually increased by 2-fold following UV exposure over 30 minutes within WT-*ssb-mTur2* cells. After this, the foci intensities remained constant for the entire 170-minute period. The foci in the RecB-deficient cells exhibited a greater intensity during the first 30 minutes, which then dropped to the level of WT-*ssb-mTur2* foci intensity after 30 minutes and persisted until the end of imaging (Fig 4D). Likewise, the intensities of large SSB clusters (monitored by mean pixel intensities measurements of SSB clusters) developed within RecB-deficient cells were greater than cluster intensities in WT-*ssb-mTur2* cells during the first 30 min (Fig 4E). After 30 min, cluster intensities between these cells remained stable (above the threshold value of 0–6100 arb. units indicated by the orange dashed line) throughout the imaging course (Fig 4E).

The size of the large SSB clusters (corresponding to large SSB features with a size >16 pixel² or >0.16 µm² as indicated by the gray dashed line) displayed by RecB-deficient strains tended to be greater than that of the SSB clusters displayed by WT-*ssb-mTur2* strains throughout the imaging period (Fig 4F), although the error bars reflecting size range overlap somewhat. Further quantification of the SSB cluster size was performed by measuring the Feret diameter (here referred to as a measurement of the longest end-to-end distance of a large fluorescent feature formed inside the cell to determine the morphology of these features). Comparing the Feret diameters of WT-ssb*-mTur2* and RecB-deficient cells also revealed that RecB-deficient cells exhibited larger SSB clusters and remained constant throughout imaging, indicating that the DNA gaps are not expanding over time (S4A Fig). The Feret diameter represents the longest distance across each SSB feature, whereas the cluster size corresponds to the total area occupied by the feature. The similarity in trends between these measurements reflects that most SSB foci and clusters are near circular in shape. The estimated SSB monomers at the end of imaging within the clusters of RecB-deficient cells (SSB monomers at 170 min = 1138 ± 709) were greater than those present within WT-*ssb-mTur2* cells (SSB monomers at 170 min = 767 ± 339). However, the number of SSB monomers within SSB foci between these cells was comparable (**Tables A and B in** S1 Text). We calculated the estimated number of SSB copies (in monomers) within these SSB features, including both foci and clusters. This was measured by dividing the mean integrated fluorescent density (the sum of the pixel intensity over all of the pixels) of SSB foci and clusters by the single-molecule mTur2 integrated density at 405 nm (ImTur2–405 = 282 arb. units), as extracted from equation 2 (For details on calculating the single-molecule mTur2 integrated intensity at 405 nm, please refer to S1 Text and S2 Table). The higher numbers of SSB in SSB clusters in the *recB* strain could reflect the significant SbcBC-dependent DNA degradation in the terminus region that has been observed in *recB* mutants [100].

### Elimination of RecFORJ pathway RMPs increases the formation of DNA-bound SSB features after UV exposure

Having established that the SSB features corresponding to SSB-bound ssDNA appear within WT *ssb-mTur2* and RecB-deficient cells after exposure to UV, we next investigated the formation of the SSB-bound ssDNA intermediates within RMP-deficient cells. We imaged *ssb-mTur2* Δ*recJ ssb-mTur2* (EAW1463), Δ*recF ssb-mTur2* (CJH0080) and Δ*recO ssb-mTur2* (CJH0081) strains to characterize the frequency of SSB foci and cluster formation along with cell morphology, mean intensity of SSB features and cluster sizes, after DNA damage.

**Fig 5:**
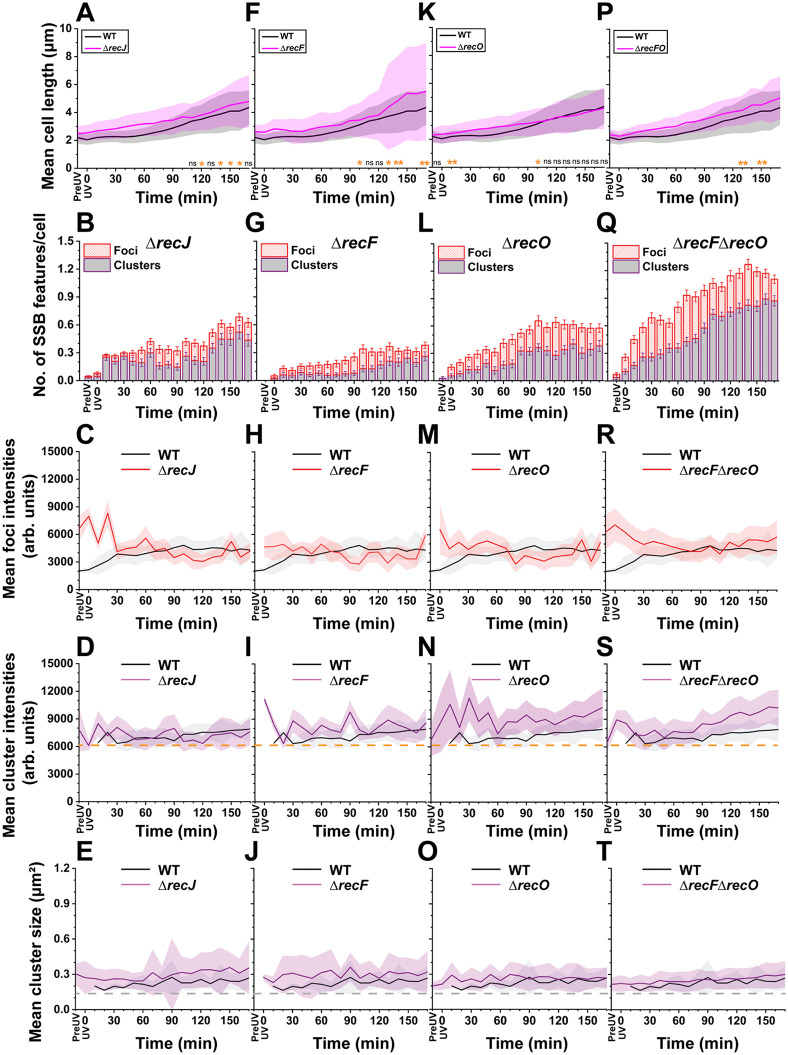
Quantification of cell length, DNA-bound SSB features (foci and clusters), foci intensity, clusters intensity and cluster size over time (170 minutes) in response to UV within *ssb-mTur2* Δ*recJ* (A-E), *ssb-mTur2* Δ*recF* (F-J), *ssb-mTur2* Δ*recO* (K-O), and *ssb-mTur2* Δ*recF* Δ*recO* (P-T) cells (N > 100 cells). Comparison of cellular length between WT-*ssb-mTur2* and **(A)** *ssb-mTur2* Δ*recJ*, **(F)** *ssb-mTur2* **Δ***recF*, **(K)** *ssb-mTur2* Δ*recO*, **(P)** *ssb-mTur2* Δ*recF*Δ*recO* cells measured over time (First row). Shaded areas represent the SD of cell length, reflecting variability within the cell population at the indicated time points. Significance is denoted (Table C in S1 Text) as follows: ns = not significant; * *p* < 0.05; ** *p* < 0.01. Time points with no ‘*’ or ‘**’ or “ns” indicate *p* < 0.001 in the figure (***); exact *p*-values and number of cells are reported in Table C in S1 Text. Quantification of ssDNA-bound SSB features (foci and clusters) displayed by; **(B)** *ssb-mTur2* Δ*recJ*, **(G)** *ssb-mTur2* Δ*recF*, l) *ssb-mTur2* Δ*recO*, **(Q)** *ssb-mTur2* Δ*recF*Δ*recO* cells over time (Second row). The red-striped box indicates SSB foci and the purple box indicates SSB clusters. The error bars represent the SEM for the number of foci or clusters formed within cells at the indicated time point. Mean intensities of SSB foci displayed by WT-*ssb-mTur2* and **(C)** *ssb-mTur2* **Δ***recJ*, **(H)** *ssb-mTur2* Δ*recF*, **(M)** *ssb-mTur2* Δ*recO*, (R) *ssb-mTur2* Δ*recF*Δ*recO* cells over time (Third row). Mean intensities of SSB clusters displayed by WT-*ssb-mTur2* and **(D)** *ssb-mTur2* Δ*recJ*, **(I)** *ssb-mTur2* Δ*recF*, **(N)** *ssb-mTur2* Δ*recO*, **(S)** *ssb-mTur2* Δ*recF*Δ*recO* cells over time (Fourth row). The orange dashed line represents the threshold intensity value (0–6100 arbitrary units) used to filter out cytosolic SSB signals for capturing SSB clusters. Mean SSB cluster sizes formed within WT-*ssb-mTur2* and **(E)** *ssb-mTur2* Δ*recJ*, **(J)** *ssb-mTur2* Δ*recF*, **(O)** *ssb-mTur2* Δ*recO*, **(T)** *ssb-mTur2* Δ*recF*Δ*recO* cells over time (Fifth row). The grey dashed line indicates the minimum cluster area (>0.16 µm2 or 16 pixel2). The shaded area in figures **(C) to (T)** (Third, fourth and fifth rows) represents the SD to emphasize the variation (intensity and size) within cell population at indicated time points.

To visualize the formation of SSB-coated ssDNA gaps within cells lacking RMPs, we first imaged Δ*recJ ssb-mTur2* (or RecJ-deficient) after UV exposure. The RecJ-deficient strains began to filament following DNA damage, and by the end of 170 min, cell length was doubled on average (Fig 5A). Like WT-*ssb-mTur2,* RecJ-deficient cells displayed few SSB features prior to UV exposure. However, after DNA damage, SSB foci and clusters increased substantially inside the cell, surpassing the effects seen in WT cells. We found that the cells displayed more SSB clusters than SSB foci by the end of 170 min following DNA damage, indicating the possible role of RecJ in ssDNA gap resolution apart from DNA gap expansion activity (Fig 5B). At the end of imaging, almost 17% of cells displayed SSB foci, and 40% displayed SSB clusters (S2C Fig). The increase in foci and clusters occurred throughout the imaging period, indicating that ssDNA gaps were generated faster than they could be resolved in the absence of RecJ. The SSB foci intensities were initially 3-fold greater than those of WT*-ssb-mTur2* foci intensities during the first 20 min which then dropped to the range of *ssb-mTur2* foci intensities after 70 min until the end of imaging (Fig 5C). However, the mean cluster intensities of RecJ-deficient cells were comparable to WT-*ssb-mTur2* cluster intensities throughout the imaging course (above the threshold value of 0–6100 arb. units indicated by the orange dashed line) (Fig 5D). Furthermore, the size of SSB clusters displayed by RecJ-deficient cells remained constant, slightly greater in size than WT-*ssb-mTur2* clusters, throughout the course of imaging (Figs 5E and S4B)

Likewise, the Δ*recF ssb-mTur2* (or RecF-deficient) cells also formed filaments with almost 3-fold increases in cell length at the end of 170 min (Fig 5F). Time-lapse imaging showed that RecF-deficient SSB-IDL fusion strains exhibited very few SSB features prior to DNA damage. However, immediately after UV exposure, cells started to display both SSB foci and clusters which then gradually increased over time (Fig 5G). At the end of 170 min, 11% of RecF-deficient cells formed SSB foci, while 24% displayed SSB clusters (S2D Fig). The SSB features displayed by RecF-deficient cells resembled a similar trend to those seen in WT-*ssb-mTur2* cells (S2A Fig). Although starting at a somewhat higher level, the SSB foci intensities of RecF-deficient cells remained stable and comparable to WT-*ssb-mTur2* foci intensities throughout the imaging course (Fig 5H). Likewise, the intensities of the SSB clusters developed immediately after UV exposure at time t = 0 were almost 2-fold higher, but they then sharply declined to the range of WT-*ssb-mTur2* until the end of 170 min (Fig 5I). While the size of SSB clusters in RecF-deficient cells was consistently larger than that in WT-ssb-mTur2 cells during the entire imaging period, there was no observed expansion in cluster size over time (Figs 5J and S4C).

The Δ*recO ssb-mTur2* (RecO-deficient) cells also began to filament after UV irradiation, nearly doubling in length by the end of the imaging (Fig 5K). Like other strains, the RecO-deficient strains also displayed few SSB features before DNA damage. However, immediately after UV exposure, both the SSB foci and clusters increased substantially (Fig 5L). The frequency of both SSB foci and clusters exhibited by RecO-deficient cells was 2-fold higher than those displayed by RecF-deficient cells (Fig 5G). At the end of imaging, more than 15% of RecO-deficient cells displayed SSB foci, whereas 36% of cells displayed SSB clusters, with nearly 0.6 features per cell (S2E Fig). The mean SSB foci intensities of RecO-deficient cells, which were initially 3-fold higher than WT-*ssb-mTur2* foci intensities after DNA damage (at t = 0), showed a downward trend after 10 min and remained constant compared to WT-*ssb-mTur2* foci intensities until the end of the imaging (Fig 5M). Similarly, the mean cluster intensities of RecO-deficient cells remained more elevated during the first 50 min, and then declined to a level slightly above WT-*ssb-mTur2* clusters intensities (Fig 5N). Furthermore, the size of the SSB clusters formed within the RecO-deficient cells remained constant and comparable to those displayed by WT-*ssb-mTur2* clusters throughout the imaging course (Figs 5O and S4D). The estimated copies of SSB monomers present within these SSB foci and clusters of RMP-deficient strains (lacking RecJ, RecF, and RecO) were not significantly different, suggesting that the absence of these proteins does not notably impact the overall composition of SSB monomers within the SSB features (See estimated SSB copy numbers within SSB foci and clusters in **Tables A and B** in S1 Text).

Collectively, these results indicate that the presence or absence of RecF does not significantly affect the initial formation of ssDNA intermediates, implying that other cellular factors can compensate for the loss of RecF under these conditions. In contrast, both RecJ and RecO proteins play essential roles in resolving SSB-bound ssDNA intermediates. Their absence results in a greater accumulation of unresolved SSB-bound ssDNA gaps, indicating that these proteins are indispensable for effective ssDNA gap resolution.

### Loss of both RecF and RecO leads to substantial generation of ssDNA intermediates

Like WT-*ssb-mTur2,* RecJ, RecF, and RecO-deficient cells, the Δ*recF* Δ*recO ssb-mTur2* (or RecFO-deficient) cells started to filament after UV exposure, and the cell length increased by more than 2-fold by the end of 170 min (Fig 5P). Prior to UV exposure, SSB features occurred at low levels within RecFO-deficient cells, with ~8% of cells displaying detectable SSB structures (approximately 4% with foci and 4% with clusters). However, after DNA damage, the SSB features, including both foci and clusters, significantly increased, exhibiting almost 3-fold higher levels than those observed in RecF-deficient cells and 2-fold higher levels than those observed in RecO-deficient cells at the end of 170 min (Fig 5Q). At the end of 170 min, 19% of RecFO-deficient cells exhibited foci, and 79% displayed clusters (S2F Fig). On average, every cell had more than one SSB feature. This suggests that the elimination of RecF and RecO function leads to an additive effect under these conditions, resulting in substantial deficits in ssDNA intermediate resolution. Moreover, the SSB foci intensities, which were initially higher after UV exposure for the first 20 min, gradually declined and remained stable at a comparable level to WT-*ssb-mTur2* foci intensities (Fig 5R). On the other hand, SSB cluster intensities within RecFO-deficient cells remained stable and slightly above the WT-*ssb-mTur2* cluster intensities throughout the imaging course (Fig 5S). Interestingly, the size of the SSB clusters within these cells was comparable to WT-ssb-mTur2 clusters and slightly smaller than those displayed by RecF-deficient and RecO-deficient cells (Figs 5T and S3E). The estimated number of SSB monomers within these SSB foci and clusters of RecFO-deficient cells was comparable to that in RMP-deficient cells. (See estimated SSB copy numbers within SSB foci and clusters in Tables A and B in S1 Text).

In general, in cells lacking RecFORJ functions, post-replication gap resolution is compromised and the gaps revealed by SSB-mTur2 tend to accumulate. However, the results seen with strains lacking different RecFORJ functions are quantitatively different. These differences are addressed in the Discussion.

## Discussion

The current work offers a range of observations and several major conclusions in relation to visually detectable SSB features in UV treated *Escherichia coli* cells growing in a minimal media. **First**, and perhaps most important, post-replication gap formation makes a major contribution to DNA repair after UV irradiation. The presence of substantial gap formation in cells lacking RecB, coupled to the sometimes dramatic increases seen when cells lack one or more of the RecFORJ components, all indicate the robust generation of post-replication gaps in response to UV irradiation. **Second**, when hundreds of UV-induced lesions are present under slow growth conditions, at least some lesions are not removed by NER for several hours. Elevated levels of SSB features reflecting UV damage persist for more than the three hours covered by these experiments (S1 Movie). **Third**, when the replisome encounters UV-generated DNA lesions, ssDNA gaps readily visualized with our labeled SSB form rapidly. In WT-*ssb-mTur2* cells, SSB features of various sizes (referred to here as SSB foci and clusters) increase in abundance shortly after UV exposure (Fig 4B). Under the conditions of these experiments (30 °C and minimal media), replication is slowed and not all cells are undergoing active replication at any given moment. The levels of SSB feature generation suggests that many, if not all active replisomes encounter UV lesions so as to trigger SSB feature generation soon after UV irradiation. **Fourth**, the SSB features observed in WT cells likely reflect the formation of ssDNA intermediates arising from double-strand break repair to some degree, as well as post-replication gap repair. Loss of RecB function, necessary for initiating the processing of double-strand breaks to form ssDNA, results in the apparent loss of perhaps a third of the SSB features observed in WT-*ssb-mTur2* cells (Fig 4C). The overall contribution of double-strand breaks to the SSB foci inventory is difficult to assess reliably due to the limited viability of *recB*^–^ cells. What is clear is that the SSB features that form in cells lacking RecB are all RecB independent, likely candidates for post-replication gaps. **Fifth,** the formation of post-replication gaps repaired by RecFORJ appears to predominate in the spectrum of gaps that appear after UV irradiation. The formation of post-replication gaps is revealed by the significant increase in unresolved SSB features upon the loss of *recO*, *recJ, recF,* or both *recO and recF* (Fig 5B, 5L and 5Q). When RecFORJ components are all present, gaps are repaired efficiently and the level of observed gaps is modest. When those components are absent, the post-replication gaps pile up. The RecFORJ functions are not essential for post-replication gap formation but are required for subsequent steps in the major resolution pathway. **Sixth**, In cells lacking one or another RecFORJ function, the accumulation of SSB features varies depending on which Rec proteins are missing, at least under these slow growth conditions. Loss of RecF has the most modest effect, perhaps reflecting its role in gap targeting. Gap repair in cells lacking RecF may have targeting mechanisms independent of RecF or gaps may be more readily repaired by other pathways such as translesion DNA synthesis. The long cell cycles under these growth conditions may allow time for RecOR to locate and target post-replication gaps even in the absence of RecF. Cells lacking RecO exhibit larger effects as the absence of RecO-mediated RecA loading leaves a greater number of gaps unresolved. Under these conditions, the effects on gap accumulation in cells lacking both RecF and RecO are additive rather than epistatic under these conditions, contrary to effects seen in many genetic studies [7]. This again may reflect the distinct functions of RecF and RecO within the larger RecFORJ pathway [64]. Gap repair is slowed or some gaps remain unresolved when RecF is missing. Loss of RecO leaves a much larger contingent of gaps unresolved. **Finally**, despite the RecF interactions with DnaN [78], a previous suggestion from our team that RecF may facilitate replisome lesion-skipping [78] is not supported by the current study (Fig 5G).

How can these results be integrated into what is known about the cellular response to UV irradiation? Multiple repair functions come into play after UV exposure, many of which can seem to act at cross purposes. Nucleotide excision repair is efficient, but typically requires multiple tens of minutes to clear most lesions from the genome [13,43–49] and at least a few lesions appear to linger for a considerable period of time [50]. Lesion-skipping will trigger recombinational repair to ameliorate gaps behind the fork, a process that entails some cellular risk. Transient topological links between the two sister chromatids behind the fork, intermediates in this recombinational DNA repair, could lead to problems in chromosome segregation at cell division [7,15]. The 20–80 min halt in DNA replication that occurs after UV irradiation [13,29,30,32,36] could prevent extensive lesion-skipping so as to avoid the potentially deleterious recombination events [13]. However, as seen here, significant lesion-skipping is occurring and post-replication gaps requiring recombinational repair are being generated. In principle, lesion-skipping could allow for continued DNA replication and replication continues for perhaps 5–10 min at 37°C [13]. But when many lesions are present, replication halts for a significant period of time, although the halt is not instantaneous [13].

Only slight variations in existing proposals are needed to fit the lesion-skipping observed here into a unified model where all of these repair systems function in a complementary fashion. Upon UV irradiation, even the lower doses of UV used here will generate hundreds of pyrimidine dimers in each genome. Nucleotide excision repair will begin to excise those lesions immediately. But NER cannot get to all of the lesions all at once. As the replisome progresses immediately after UV, lesion-skipping may be the default outcome for encounters with intact UV lesions not yet addressed by NER. Many such intact lesions are likely to be encountered in the first few seconds or minutes in cells where replisomes are active, rapidly generating multiple post-replication gaps. Eventually (within several minutes), the replisome encounters either a template strand discontinuity created by ongoing NER or a different lesion/protein complex that cannot be bypassed, resulting in replisome collapse. With thousands of lesions present in a cell with functioning NER, this collapse is inevitable. Within those early minutes, replisome collapse produces a halt in DNA replication over the entire cell population. However, the postulated quick burst of lesion-skipping that preceded the halt serves an important purpose. Active replication forks [52] and the generation of significant levels of ssDNA is required for SOS induction [52,101–104]. The resultant formation of RecA filaments in post-replication gaps initiates gap repair and, perhaps more important, creates the essential conditions required for rapid SOS induction. SOS in turn increases NER components and otherwise hastens genomic repair. In line with work cited above, a burst of post-replication gaps, formed immediately after UV irradiation, may thus be the primary source of the ssDNA that triggers SOS. In further support of this idea, we note that induction of the SOS response is substantially delayed in cells lacking RecFORJ functions [105,106]. An apparent quick burst of post-replication gap formation is more readily seen under conditions of more rapid growth in the accompanying study [107].

This study presents a first look at the types of SSB features that develop in *E. coli* cells under slow growth conditions after exposure to UV light. We employed this approach to exert control over the development of fluorescent SSB features within cells. Under rapid growth conditions (37 °C and rich media), the simultaneous emergence of multiple SSB features can complicate accurate quantification. Complementary results obtained under conditions of more rapid growth and different visualization protocols are described in the accompanying report [107]. The effects of RecA and RecJ are also better considered there. That report both extends the current one while also helping to ensure that the basic patterns we observe are not artifacts of the experimental protocol. While the fluorescent SSB features developed under the current conditions are significantly fewer compared to those observed in previous studies [79,93], the lower background allows us to more readily observe the development of SSB features formed within wild-type cells and cells lacking either RMPs or DSBR proteins after UV irradiation.

Along with many other systems as noted in the Introduction, RecBCD-mediated double strand break repair is required for full resumption of replication after UV irradiation [34]. RecBCD is probably most important for amelioration of problems encountered near the termination of replication [34,108–110]. The UV sensitivity of cells lacking RecB is extreme and many of the cells we are observing will eventually die. RecB-deficient cells showed larger average sizes of SSB clusters compared to wild type and RMP-deficient cells along with a greater range of sizes. We speculate that, as a level of double strand breaks is still present and RecJ function is involved in a backup repair path relative to RecBCD, processing of DSBs by RecJ may produce larger ssDNA intermediates than is the case for RecBCD (Fig 4F). Our results also show that the RecB-deficient cells did not form filaments after DNA damage, as observed by Cherry et al. [79], suggesting that the SOS response was not activated. Recent genetic studies have that the deletion of RecB in *E. coli* reduces the ability of cells to activate SOS [111]. The filamentation of bacterial cells is a hallmark of the SOS response, which allows the cell to survive in a stressful environment by enabling them to continue replicating their DNA and repairing any DNA damage while avoiding cell division and the potential creation of daughter cells with damaged DNA [63,101,112–115]. Overall, the absence of the SOS response, coupled with the formation of fewer SSB features and larger cluster sizes by RecB-deficient cells, suggests that RecB plays a significant role in the observable metabolism of UV damage.

The large increase in unresolved gaps revealed by SSB foci in the absence of RecFORJ system functions indicates that most SSB features are likely attributable to post-replication gaps formed as a result of replisome bypassing the UV lesions. When the entire RecFORJ system is intact, the level of SSB features seen is relatively low as the gaps are efficiently resolved. The gaps would disappear in the cells under observation as soon as RecA was loaded and the SSB displaced. When RecA is not loaded efficiently due to the absence of RecJ, RecF, and/or RecO, the resolution of post-replication gaps is compromised. This is evident from our study, where the accumulation of SSB features increases following DNA damage and takes a longer time to disappear compared to wild-type cells (Figs 4B, 5B, 5G, 5L and 5Q). Our observations highlight the critical roles of RecJ and RecO in resolving post-replication gaps and speaks to the high frequency of replisome lesion-skipping and post-replication gap formation after UV irradiation [7]. While RecJ facilitates the formation of SSB-bound ssDNA intermediates by expanding the gaps [15,20], RecO subsequently facilitates the removal of SSB proteins from these gaps as RecA is loaded [64,73,81,116].

RecF interacts with DnaN, driving speculation that RecF might facilitate replisome lesion skipping and post-replication gap formation via this interaction [78]. Deletion of *recF* resulted in the formation of SSB features at levels comparable to WT-*ssb-mTur2* cells. Thus, RecF is not required for the generation of many of the SSB features observed and may not be required for the generation of any of them. The levels of SSB features seen in *ΔrecF* cells are substantially less than those observed in Δ*recJ* and especially Δ*recO* cells. This might suggest that some SSB features are created with the aid of RecF, as the number of observable gaps increases when RecF (generally involved in gap targeting) is present but subsequent steps carried out by RecO cannot be completed. However, deletion of both *recF* and *recO* leads to a more significant increase in the formation of the SSB features (Fig 5Q), with the *recF* and *recO* results approximately additive. Under these conditions, loss of the RecF and RecO functions together exhibits apparent synergism rather than epistasis with respect to the generation of gaps. This finding suggests that (a) RecF is truly unimportant in replisome lesion skipping (it is not present in the *recFO* cells and observable gaps are increasing), and (b) the role of RecF in post-replication gap repair may be more complex than considered to date. In the double mutant, at least some gap resolution events that are possible in each of the *recF* and *recO* single mutants are not occurring under slow growth conditions. The results may reflect undocumented effects of RecF and/or RecO with alternative gap resolution pathways such as translesion DNA synthesis [117], or the RecA-independent template switch path [24,118].

Elevated levels of SSB features, mainly large SSB clusters reflective of UV damage, persist for the entire period of observation in these experiments, nearly three hours. This is true even in WT cells (Movie A in S2 Movie). In those WT cells, these are generally not the same SSB foci persisting over this time period in WT cells, but rather a steady state in which replisomes are encountering lesions anew and forming new gaps as others are resolved. This prolonged persistence highlights the considerable challenge that UV-induced DNA damage poses to cellular repair mechanisms. Nucleotide excision repair is efficient [13,43–49] but is unable to completely rid the genome of UV lesions within this timescale when hundreds of such lesions are introduced, at least under these slow growth conditions. The effects of lesion skipping is especially evident in RecFORJ-deficient cells, where SSB features continually accumulate within cells for almost 3 hours (Fig 5B, 5G, 5L and 5Q), especially when both RecF and RecO are absent.

While our work has enabled the establishment of relationships between ssDNA-bound SSB features and their dependence on repair factors involved in the initial steps of recombinational DNA repair at longer timescales, it is still limited by the inability to capture SSB dynamics at shorter timescales. A recent study by Cherry et al. reported highly dynamic SSB features displayed by cells on a low-minute timescale resolution [79]. These features exhibited various transformations, such as brightening, dulling, merging, and splitting, occurring on a microsecond timescale [79]. Similar transformations of SSB features were observed in our study (S1 Movie), suggesting their constant changes in response to DNA damage during repair processes. However, it is difficult to follow individual foci for extended periods in our study due to its longer timescale (recorded images at every 10-minute interval for 170 minutes). During this period, the initially formed SSB foci may have reorganized multiple times. Additionally, the longer time intervals between image recordings in our study may have limited our ability to detect SSB foci representing ssDNA intermediates that could have formed and dispersed inside cells. This limitation also explains why a lower percentage of WT-*ssb-mTur2* cells may have displayed SSB features compared to RecFORJ-deficient cells, which lack essential factors to resolve ssDNA intermediates, thereby leading to the accumulation of more SSB-bound features that persisted over several hours and could be captured at longer time intervals. (S2C, S2E and S2F Fig). Direct ori:ter measurements would be valuable addition for future studies aimed at quantifying replication fork abundance under microfluidic imaging conditions.

The present work is an early effort to directly define features of DNA metabolism after UV irradiation in *E. coli*. These and closely related methods [79,107] combined with the many DNA repair mutants available, have potential for providing many additional new insights into these repair processes.

## Materials and methods

### E. coli strains used in this study

A complete list of the strains used in this study appears in S1 Table. EAW1169 (*ssb-mtur2*), EAW1219 (*ssb-mtur2* Δ*recB*), and CJH0080 (Δ*recF ssb-mtur2*) have been described previously [78,79,93]. CJH0081 (Δ*recO ssb-mtur2*) and CJH0082 (*ΔrecO* Δ*recF ssb-mtur2*) were created by transducing the *ssb-mtur2::kanR* allele into EAW114 (Δ*recO*) [64] and EAW668 (Δ*recO* Δ*recF*), respectively, using standard P1-transduction protocols. A Δ*recJ* derivative of EAW1169 (EAW1463) was created by replacing the Δ*recJ* gene with a kanamycin cassette flanked by FRT sites with the Datsenko-Wanner protocol [119].

### Growth curves

The growth of *E. coli* strains (EAW1169, EAW1219, EAW1463, CJH0080, CJH0081 and CJH0082) in minimal medium (with UV or without UV exposure) was assessed. Strains were cultured in M9 medium (48mM Na2HPO4, 22 nM KH2PO4, 8.6mM NaCl, 18.6mM NH4Cl, 20mM Glucose, 2mM MgSO4 and 0.1mM CaCl2) at 30°C with shaking at 800 rpm (ThermoMixer C, Eppendorf). Saturated overnight cultures were reset 1:10–1 mL in 2 mL Eppendorf tubes. Absorbance at 600 nm (OD_600_) was measured (in triplicate) every 2 hours on the spectrophotometer (Biophotometer, Eppendorf). OD_600_ values were plotted over time to determine the growth phase of each strain.

### UV-survival assay

All cells expressing *ssb-mtur2* (both WT and recombination deficient cells) were tested for sensitivity to UV. Cells were grown in M9 medium at 30 °C with shaking at 800 rpm. Cell culture in mid-exponential phase was serially diluted in phosphate buffered saline (PBS 1X, pH 7.4) before spot plating on 30 mL LB (Luria-Bertani) plates in replicates. Each set of plates was submitted to either 5, 10, or 15 J/m2 UV light using a crosslinker (Spectrolinker XL-1000). Plates were kept in the dark and incubated at 30 °C for 32 hours prior to imaging.

### Preparation of APTES-functionalized coverslips

3-aminopropyltriethoxysilane (APTES) functionalized glass slides were prepared as previously described [120]. First, glass slides (Marienfeld, Deckglaser, 24 × 50 mm No. 1.5 and 24 × 55 mm No. 1 Germany) were sonicated for 20 min in a 4M KOH solution at room temperature to clean the surface. Clean slides were thoroughly rinsed with Milli-Q water, then incubated in a 5% (v/v) solution of APTES in Milli-Q water for 5–10 min at room temperature. After that, slides were sonicated in 100% ethanol for 20 seconds before being rinsed with Milli-Q water. The functionalized coverslips were dried under a stream of nitrogen and stored in an airtight container.

### Flow-cell assembly

Home-built quartz-based flow cells, described previously by Ghodke et al. [120], were used in this study to perform time-lapse imaging for 3 hours. Briefly, a 30–45 cm inlet and outlet tubing (BTPE-60, Walker Scientific, AUS) was glued (BONDiT B-482 epoxy, Reltek, USA) to a quartz piece (Proscitech, Australia). An APTES -(3-Aminopropyltriethoxysilane)-functionalized coverslip (Marienfeld, Deckglaser, 24 × 50 mm No. 1.5, Germany) was then attached to the quartz piece (Proscitech, Australia) using a double-sided sticky tape (970XL ½ X 36yd, 3M) to create a channel. The interfaces of quartz/coverslip and quartz/tubes were further sealed with epoxy (Parafix, AUS).

### Cell culture for imaging

A DMSO stock of cells stored at -80°C was revived by inoculating a 1 mL culture of M9 minimal medium overnight at 30°C with constant shaking at 800 rpm on an Eppendorf Thermomixer C (Eppendorf, Australia). For all imaging experiments, cells were then grown at 30°C for 5–8 hours to exponential phase (OD_600_ range 0.2-0.3) in the M9 minimal media. Cells were loaded onto the APTES-treated flow-cell using a syringe pump (Adelab Scientific, Australia) at a flow rate of 30 µL/min. Once sufficient cells (N > 50 cells) were adsorbed on the surface of the flow cell, fresh aerated medium without any cells was circulated via the inlet tubing and the loosely attached cells were flushed off. Imaging was performed on a heated stage at 30°C (to maintain cell growth). *In situ* UV-irradiation was used to deliver UV light to cells at 10 J/m^2^ via a UV pen-ray light source (UVPA90-0012-01, 11SC-1, 254 nm, Upland CA, USA) with a G-275 filter allowing transmission of 256 nm UV [120]. The exposure time and the required UV dosage were determined by measuring the UV flux with a UVX radiometer (UVP, Australia). Additional double-imaging of WT *ssb-mtur2* strains (EAW1169) cells grown in the M9 minimal medium and EZ-rich medium (supplemented with 0.2% (v/v) glucose) either at 30°C or 37°C, was performed to compare the effects of temperature and growth medium on the formation of fluorescent SSB features inside the cell.

### Single-molecule live-cell imaging

Live-cell imaging was performed on a home-built inverted fluorescence microscope (Nikon Eclipse-Ti) equipped with a 1.49 NA 100X objective and a 512 x 512 pixel^2^ EM-CCD camera (Andor iXon Life, EMCCD) to record the images. This microscope was equipped with 405 (OBIS, Coherent, CA), 488 (Sapphire, Coherent, CA), 514 (Sapphire, Coherent, CA), 568 (Sapphire, Coherent, CA) and 647 (OBIS, Coherent, CA) nm lasers. Time-lapse imaging of the cells was carried out to characterize the fluorescent signals in the wild-type and recombination mutant cells by exciting the cells with 405 nm (power density: 107 W/cm^2^) OBIS Coherent 200 mW laser illumination in highly inclined laminated optical sheet (HILO) mode. Pre-UV images (before exposing the cells to UV) and the post-UV images at 30–45 distinct locations were recorded at intervals of 10 min for a total of 170 min.

Live-cell imaging control experiment was also performed to visualize the effects of growth medium and temperature on the development of fluorescent features within cells at 30 °C and 37 °C using a 405 nm laser (power density: 100 W/cm^2^) and a 458 nm laser (power density: 10 W/cm^2^). This was performed using a second custom-built wide-field fluorescence microscope equipped with a Nikon Eclipse Ti-2 body, a 512 x 512 pixel^2^ EM-CCD camera (C9100-13, Hamamatsu, Japan), 405 nm (OBIS, Coherent, USA), 458 nm (Sapphire, Coherent, USA), and 514 nm (Sapphire, Coherent, USA) and 568 nm (Sapphire, Coherent, USA) lasers. NIS-Elements software (Nikon, Japan) was used to control the microscopes [120]. Time-lapse imaging of the bacterial cells grown in M9 minimal and EZ-rich media at 30 °C was recorded every 10 min for 170 min by illuminating with 405 nm (power density: 100 W/cm^2^) and 458 nm (power density: 10 W/cm^2^) lasers in HILO mode. Images of cells grown in M9 minimal and EZ-rich media at 37 °C were captured from 10 distinct locations at 2-minute intervals for 28 min. All cells were imaged without exposure to UV.

### Image processing

Image processing was performed in Fiji version 1.51n using custom macros and a s*ingle-molecule biophysics* plug-in as previously described [79]. Briefly, the raw ND2 images were first converted to TIF format using a custom macro *Process Raw Images*. Image flattening and background corrections were performed to reduce the excitation inhomogeneity and background signal from the flow cell surface [121]. Following image processing, manual cell outlines were drawn in a MicrobeTracker 0.937 MATLAB plug-in [122] to select in-focus, non-overlapping cells. In a well-established analytical pipeline, the ROIs were then further analyzed using custom-written code generated using Fiji software [120] to measure different parameters within the cells, including cell length, number of SSB foci per cell, mean intensity of SSB foci and foci area.

### Characterization of fluorescent SSB signals inside cells

A custom Fiji macro, Particle Analysis Suite 4, was used to characterize fluorescent SSB features inside cells as previously described [79]. Manually drawn cell outline regions of interest (ROIs) were exported from MicrobeTracker 0.937 and used to measure mean cell fluorescence intensities. Bound SSB features were then characterized as either diffraction-limited foci (termed as “foci”) or large clusters of indistinguishably overlapping foci (termed as “clusters”). Thresholding with a 16-bit intensity range of 0 – 6100 and an 8-bit intensity range of 175 – 255, was used to identify SSB clusters ROIs as previously described [79]. ROIs not contained within the boundary of cell outlines and with an area less than or equal to 16 sq. pixels were discarded. SSB diffraction limited foci were detected via Peak Fitter from the single-molecule biophysics Fiji plug-in [79]. Foci were identified as diffraction-limited spots with an area of 16 sq. pixels (irrespective of the threshold intensity) located on top of the cytosolic background. Only SSB foci located within the bounds of cell ROIs but outside the bounds cluster ROIs were used for analysis.

### Measurement of SSB copy numbers inside the cell

The SSB copy number within the bacterial cells was measured previously by Cherry et al. using low-copy expression of fluorescent SSB from an arabinose-inducible plasmid [79]. The mTur2 single-molecule integrated density of 176 arbitrary units (95% confidence interval: 160–208) derived from the photobleaching experiment by Cherry et al. was used in this study for calculating the estimated SSB copy number within SSB features of the cells. Briefly, the WT-*ssb-mTur2* cells (EAW1169) supplemented with EZ-rich medium (at comparable growth conditions as previously in Cherry et al.) were imaged with 458 nm and 405 nm lasers. The imaging setup for the 458 nm laser was kept similar to the imaging condition used by Cherry et al. [79], whereas the imaging setup for the 405 nm laser was unchanged. Analysis of the cellular fluorescence was performed as follows: The cell images (captured using 458 nm and 405 nm lasers) were first background corrected. Next, the ‘corrected mean integrated density’ for each cell (N = 40 cells) imaged at 458 nm was calculated from the signal contained within ROIs representing the outline of the cells. Following this, the corrected mean integrated density of the cells was then divided by the single-molecule mTur2 integrated density (previously estimated to be 176 arb. units by multiplying the single molecule mean pixel intensity with pixel area of a foci [79]) to calculate the estimated number of SSB molecules (Equation 1).


$$\text{N }=\;{\text{I}}_{\text{458}}/{\text{I}}_{\text{mTur2}-\text{458}}$$ Equation 1

Where N = number of SSB, I_458_ = Mean integrated density of the cells at 458 nm, I_mTur2–458_ = single-molecule integrated density of the mTur2 at 458 nm.

Since the cells were imaged simultaneously in both channels (458 nm and 405 nm), the estimated SSB cell copy numbers obtained *via* imaging with a 458 nm laser are expected to be the same for the cells imaged with a 405 nm laser. Therefore, the SSB cell copies derived from I_458_ (mean integrated densities of cells imaged with a 458 nm laser) correspond to the SSB cell copies at I_405_ (mean integrated densities of cells imaged with a 405 nm laser).

$$\text{SSB copy number at }{\text{I}}_{\text{458}}\;(\text{EZ})\;=\text{ SSB copy number at }{\text{I}}_{\text{405}}\;(\text{EZ})$$

By dividing the mean integrated densities of cells imaged with a 405 nm laser (I_405_) with the SSB cell copy number obtained from equation 1, we can then extract the mean single-molecule mTur2 integrated density of 405 nm cell images (**see** S1 Text and S2 Table f**or the calculation of I**_**mTur2–405**_).


$${\text{I}}_{\text{mTur2}-\text{405}}={\text{I}}_{\text{4}\text{0}\text{5}}/\text{N}$$ Equation 2

Where *I*_*mTur2–405*_ = single-molecule integrated density of the mTur2 at 405 nm, *N* = number of SSB, *I*_*405*_ = Mean integrated density of the cells at 405 nm.

Using the single-molecule mean integrated density (ImTur2–405) obtained from equation 2, we can then calculate the estimated total SSB cell copy number within the fluorescent SSB features of the WT and recombination-deficient cells grown in the M9 minimal medium (**Refer to Tables A and B i**n S1 Text **for the calculation of estimated SSB copy numbers within SSB features formed inside cells**).

The SSB counts presented here are estimates derived from mean integrated fluorescence intensities and are intended to provide relative comparisons between strains and conditions rather than absolute molecular counts. Owing to the limitations of single-plane imaging and the need to minimize photobleaching during time-lapse acquisition, three-dimensional or single-particle tracking approaches were not applied in this study but represent an important scope for future quantitative analyses.

### Data and statistical analysis

Dynamics of SSB foci and clusters (including percentage changes in the number of foci and clusters, foci and cluster intensities, and cluster sizes) were quantified from time-lapse imaging data acquired across 19 time points (pre-UV to 170 min post-UV exposure) in WT and recombination-deficient strains (Δ*recB*, Δ*recJ*, Δ*recF*, Δ*recO*, and Δ*recFO*). Raw data, consisting of aggregated counts of cells displaying SSB foci and clusters per time point, were stored in an Excel file.

Data analysis was performed using custom Python scripts in Jupyter Notebook (Anaconda3 environment) with the pandas, numpy, and scipy.stats libraries. Percentage changes in SSB foci or cluster number, intensity, and cluster size relative to the WT strain were calculated. Percentage changes were not reported when the WT mean was zero and the mutant mean was non-zero, to avoid undefined values.

Statistical comparisons between WT and each mutant strain at corresponding time points were conducted using Welch’s unpaired t-test to accommodate potentially unequal variances. Statistical significance was defined as p < 0.05 and indicated as * (p < 0.05), ** (p < 0.01), or *** (p < 0.001). All calculated metrics, including means, SD, SEM, percentage changes, and exact p-values, were exported as CSV files to ensure reproducibility.

## Supporting information

S1 Fig UV survival assay and growth curve of SSB-IDL fusion expressing *E. coli* grown in M9 at 30°C. (A) Spot plates showing sensitivity to UV as indicated by WT-*ssb-mTur2*, Δ*recB ssb-mTur2* and Δ*recJ ssb-mTur2* cells. (B) Spot plates showing sensitivity to UV as indicated by *ssb-mTur2*, Δ*recF ssb-mTur2* and Δ*recO ssb-mTur2 and* Δ*recF* Δ*recO ssb-mTur2.* Strains without SSB fusion (WT, Δ*recB,* Δ*recJ,* Δ*recF,* Δ*recO* and Δ*recF* Δ*recO*) were used as controls. (C) Growth curve of SSB-IDL fusion-expressing strains in M9 medium at 30°C without UV exposure. Error bars represent standard deviation. (D) Growth curve of SSB-IDL fusion-expressing strains in M9 medium at 30°C after UV exposure (10 J/m²). Error bars represent standard deviation.
(TIF)

S2 Fig Percentage of SSB-IDL fusion strains displaying fluorescent SSB foci (red striped box) and SSB cluster (purple box) over time within the cell population (N > 100). Percentage of (A) WT *ssb-mTur2*, (B) Δ*recB ssb-mTur2*, (C) Δ*recJ ssb-mTur2* (D) Δ*recF ssb-mTur2,* (E) Δ*recO ssb-mTur2* and (F) Δ*recF*Δ*recO ssb-mTur2* cells displaying SSB foci and cluster over time. The error bar represents the standard error of the mean number of cells forming SSB features (foci and clusters) at the indicated time points.
(TIF)

S3 Fig Quantification of pre-UV SSB foci in WT *ssb-mTur2* strains under different growth conditions and comparison of WT *ssb-mTur2* cell length with and without UV exposure in M9 minimal media. (A) Percentage of WT *ssb-mTur2* strains displaying fluorescent SSB features without UV exposure under different growth conditions at the initial time point (t = 0 min). The cells were grown in M9 minimal (n = 51 cells) or EZ-rich (n = 50 cells) media at either 30 °C or 37 °C. Cell images were recorded using the 405-nm (magenta) and 458-nm (cyan) lasers. Error bars represent the SD, reflecting variability in SSB feature formation within cell population. (B) Comparison of WT *ssb-mTur2* cell length with UV exposure (indicated by the magenta line) and without UV exposure (indicated by the black line) after 405 nm laser excitation. Shaded areas represent the standard error of the mean cell length at the indicated time points.
(TIF)

S4 Fig Measurement of Feret’s diameter of SSB clusters formed inside WT *ssb-mTur2*, (A) *ssb-mTur2* Δ*recB*, (B) *ssb-mTur2* Δ*recJ* (C) *ssb-mTur2* Δ*recF,* (D) *ssb-mTur2* Δ*recO* and (E) *ssb-mTur2* Δ*recF* Δ*recO* cells, over time. The shaded area represents the standard deviation to emphasize the variation of SSB clusters size within cell population at indicated time points.
(TIF)

S5 Fig Quantification of percentage change in SSB foci intensities in WT and recombination-deficient strains over time following UV exposure. Mean SSB foci intensities and percentage change in mutant strains relative to WT cells at the indicated time points following UV exposure. Comparisons are shown for WT and (A) Δ*recB*, (B) Δ*recJ*, (C) Δ*recF*, (D) Δ*recO*, and (E) Δ*recFO* strains. Percentage change represents the difference in mean foci intensity of each mutant relative to WT at the corresponding time point. (F) Overall mean SSB foci intensities per strain, averaged across the 170-min time course. Error bars represent the SD, reflecting variability in foci intensity across individual cells at each time point. Statistical significance denotes comparisons between mutant and WT strains at the same time point and is indicated as follows: * *p* < 0.05; ** *p* < 0.01; *** *p* < 0.001. Exact *p*-values and the number of cells displaying foci for panel A-E are provided in Table D in S1 Text, and those for panel F are provided in Table I in S1 Text.
(TIF)

S6 Fig Quantification of percentage change in SSB cluster intensities in WT and recombination-deficient strains over time following UV exposure. Mean SSB cluster intensities and percentage change in mutant strains relative to WT *ssb-mTur2* cells at the indicated time points following UV exposure. Comparisons are shown for WT and (A) Δ*recB*, (B) Δ*recJ*, (C) Δ*recF*, (D) Δ*recO*, and (E) Δ*recFO* strains. Percentage change represents the difference in mean cluster intensity of each mutant relative to WT at the corresponding time point. (F) Overall mean SSB cluster intensities per strain, averaged across the 170-min time course. Error bars represent the SD, reflecting variability in cluster intensity across individual cells at each time point. Statistical significance denotes comparisons between mutant and WT strains at the same time point and is indicated as follows: * *p* < 0.05; ** *p* < 0.01; *** *p* < 0.001. Exact *p*-values and the number of cells displaying clusters for panel A-E are provided in Table E in S1 Text, and those for panel F are provided in Table I in S1 Text.
(TIF)

S7 Fig Quantification of percentage change in SSB cluster size in WT and recombination-deficient strains over time following UV exposure. Mean SSB cluster size and percentage change in mutant strains relative to WT cells at the indicated time points following UV exposure. Comparisons are shown for WT and (A) Δ*recB*, (B) Δ*recJ*, (C) Δ*recF*, (D) Δ*recO*, and (E) Δ*recFO* strains. Percentage change represents the difference in mean cluster size of each mutant relative to WT at the corresponding time point. (F) Overall mean SSB cluster size per strain, averaged across the 170-min time course. Error bars represent the SD, reflecting variability in cluster size across individual cells at each time point. Statistical significance denotes comparisons between mutant and WT strains at the same time point and is indicated as follows: * *p* < 0.05; ** *p* < 0.01; *** *p* < 0.001. Exact *p*-values and the number of cells displaying clusters for panel A-E are provided in Table F in S1 Text, and those for panel F are provided in Table I in S1 Text.
(TIF)

S8 Fig Quantification of the percentage change in the number of SSB foci in WT and recombination-deficient strains over time following UV exposure. The number of SSB foci per cell and the percentage change in mutant strains relative to WT cells are shown at the indicated time points following UV exposure. Comparisons are shown between WT and (A) Δ*recB*, (B) Δ*recJ*, (C) Δ*recF*, (D) Δ*recO*, and (E) Δ*recFO* strains. Percentage change represents the difference in the mean number of SSB foci per cell in each mutant relative to WT at the corresponding time point, with increases indicated by green upward arrows and decreases indicated by red downward arrows. Error bars represent the SD, reflecting variability in SSB foci numbers across individual cells at each time point. Statistical significance denotes comparisons between mutant and WT strains at the same time point and is indicated as follows: *p* < 0.05; *p* < 0.01; *p* < 0.001. Exact *p*-values and the number of cells analyzed are provided in Table G in S1 Text.
(TIF)

S9 Fig Quantification of the percentage change in the number of SSB clusters in WT and recombination-deficient strains over time following UV exposure. The number of SSB clusters per cell and the percentage change in mutant strains relative to WT cells are shown at the indicated time points following UV exposure. Comparisons are shown between WT and (A) Δ*recB*, (B) Δ*recJ*, (C) Δ*recF*, (D) Δ*recO*, and (E) Δ*recFO* strains. Percentage change represents the difference in the mean number of SSB clusters per cell in each mutant relative to WT at the corresponding time point, with increases indicated by green upward arrows and decreases indicated by red downward arrows. Error bars represent the SD, reflecting variability in SSB cluster numbers across individual cells at each time point. Statistical significance denotes comparisons between mutant and WT strains at the same time point and is indicated as follows: *p* < 0.05; *p* < 0.01; *p* < 0.001. Exact *p*-values and the number of cells analyzed are provided in Table H in S1 Text.
(TIF)

S1 Movie Time-lapsed brightfield (Top) and 405 nm (bottom) movies of the indicated SSB-IDL fusion expressing strains following UV exposure (10 J/m2) over time. The SSB clusters (with an area >16 pixel²) that develop over time are perfectly captured (small yellow outline inside the yellow cell outlines) by thresholding the 16-bit intensity range to 0–6100 arbitrary units (au) and the 8-bit intensity range to 175–255 au for all processed image stacks using Fiji’s default thresholding algorithm. Scale bar represents 2 µm.
(PPTX)

S2 Movie Time-lapsed brightfield (Top), 405 nm (Middle) and 458 nm (Bottom) movies of the WT-sb-mTur2 strain (EAW1169) grown in (A) M9 minimal at 30 °C, (B) EZ-rich medium at 30 °C, (C) M9 minimal at 37 °C and (D) EZ-rich medium at 37 °C. Images of cells grown in M9 minimal and EZ-rich media at 37 °C were recorded at every 2-minute interval for 28 min whereas, images of cells grown in either M9 minimal or EZ-rich medium at 30 °C were recorded at every 10-minute interval for 170 minutes. All cells images were without exposing to UV. Scale bar represents 2 µm.
(PPTX)

S1 Table *E.coli* strains used in this study. (DOCX)

S2 Table Table list showing the integrated density and SSB copy number of *ssb-mTur2* cells (EAW1169) imaged with 458 nm and 405 nm lasers. (DOCX)

S1 Text Calculations for single-molecule mTur2 integrated intensity at 405 nm to determine SSB cell copy number. (DOCX)
