# Supplementary figures and images for: Spatiotemporal characterization of single-stranded DNA Intermediates after UV Irradiation: I: Post-replication gaps formed during slow growth
Source: PLoS Genet. 2026 May 14;22(5):e1012109. doi: 10.1371/journal.pgen.1012109 (PMC13175387; doi:10.1371/journal.pgen.1012109)

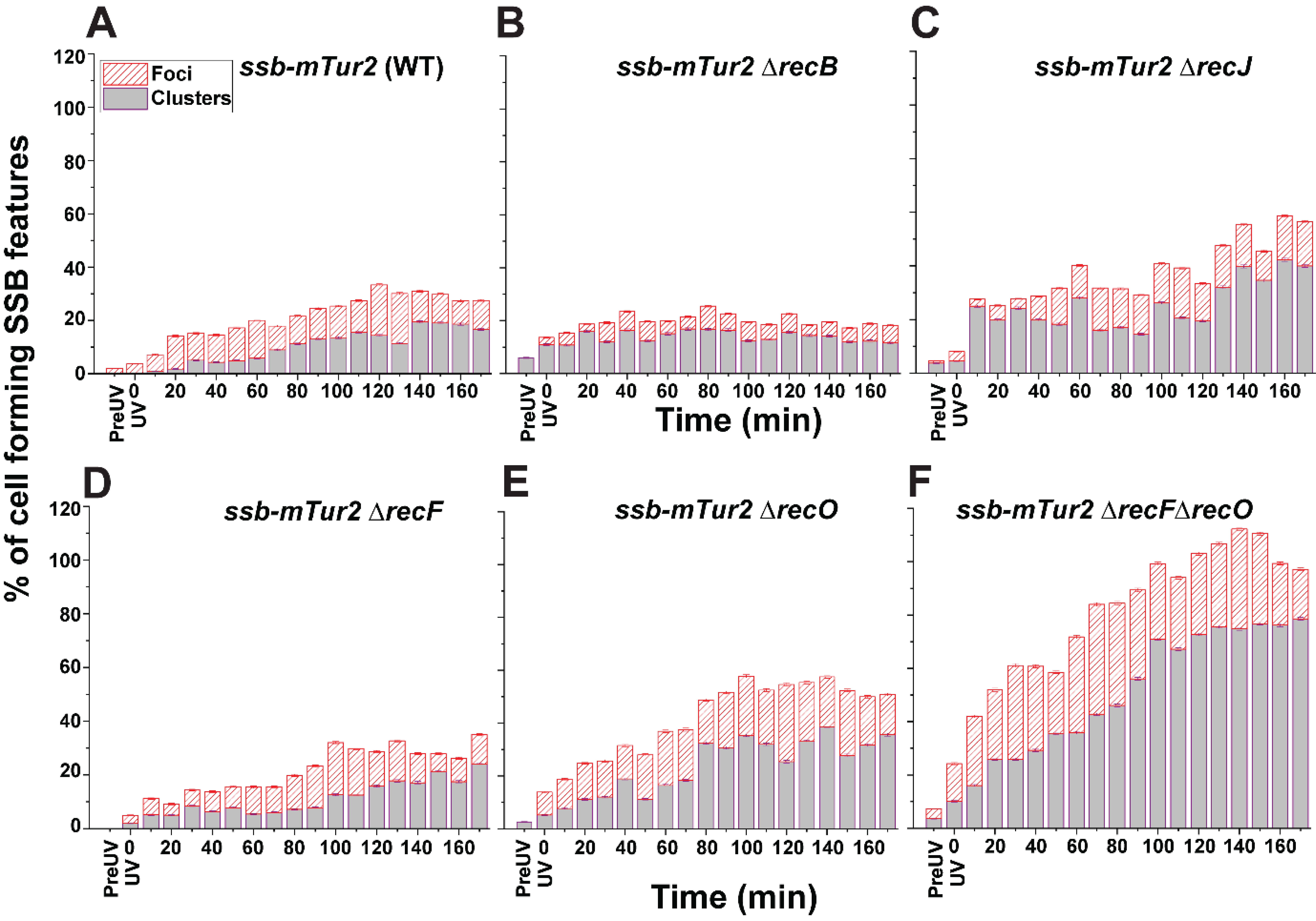

Supplement: S2 Fig — Percentage of (A) WT ssb-mTur2, (B) ΔrecB ssb-mTur2, (C) ΔrecJ ssb-mTur2 (D) ΔrecF ssb-mTur2, (E) ΔrecO ssb-mTur2 and (F) ΔrecFΔrecO ssb-mTur2 cells displaying SSB foci and cluster over time. The error bar represents the standard error of the mean number of cells forming SSB features (foci and clusters) at the indicated time points. (TIF) [file pgen.1012109.s002.tif]

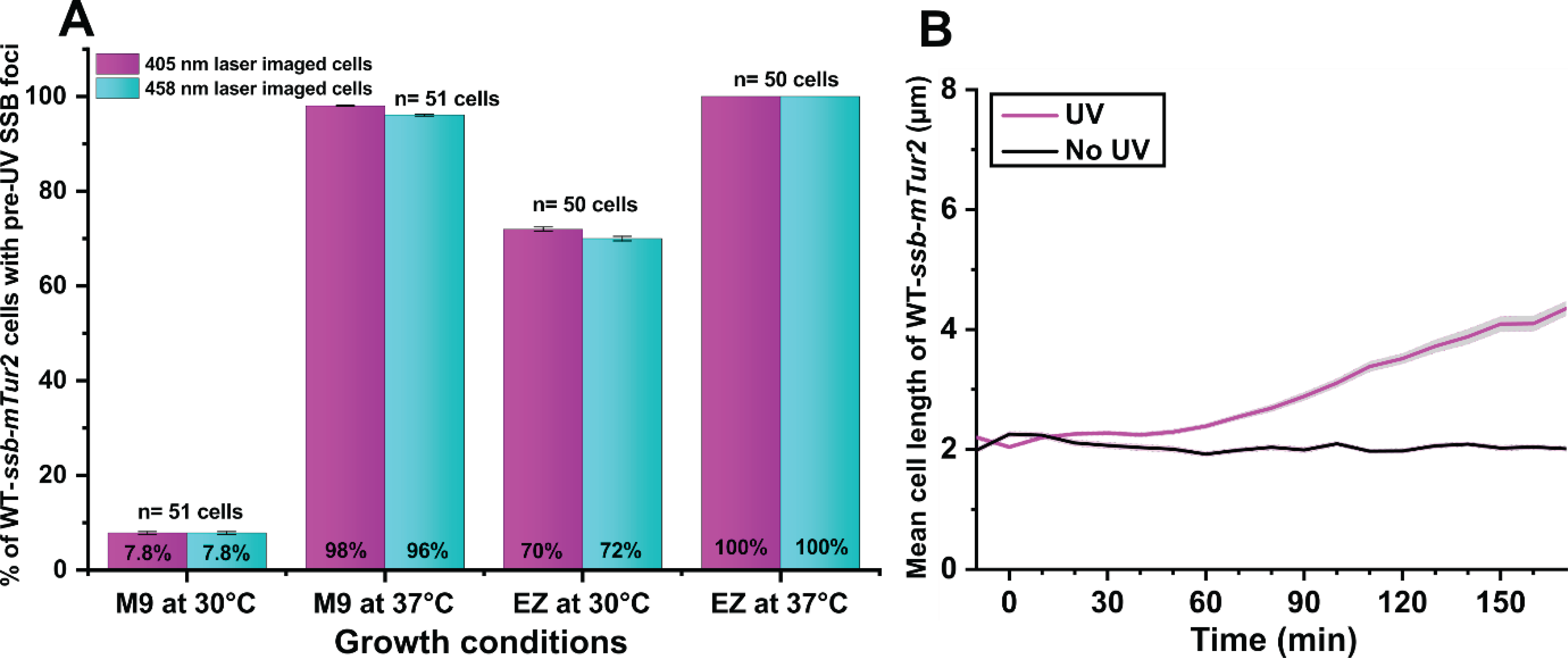

Supplement: S3 Fig — (A) Percentage of WT ssb-mTur2 strains displaying fluorescent SSB features without UV exposure under different growth conditions at the initial time point (t = 0 min). The cells were grown in M9 minimal (n = 51 cells) or EZ-rich (n = 50 cells) media at either 30 °C or 37 °C. Cell images were recorded using the 405-nm (magenta) and 458-nm (cyan) lasers. Error bars represent the SD, reflecting variability in SSB feature formation within cell population. (B) Comparison of WT ssb-mTur2 cell length with UV exposure (indicated by the magenta line) and without UV exposure (indicated by the black line) after 405 nm laser excitation. Shaded areas represent the standard error of the mean cell length at the indicated time points. (TIF) [file pgen.1012109.s003.tif]

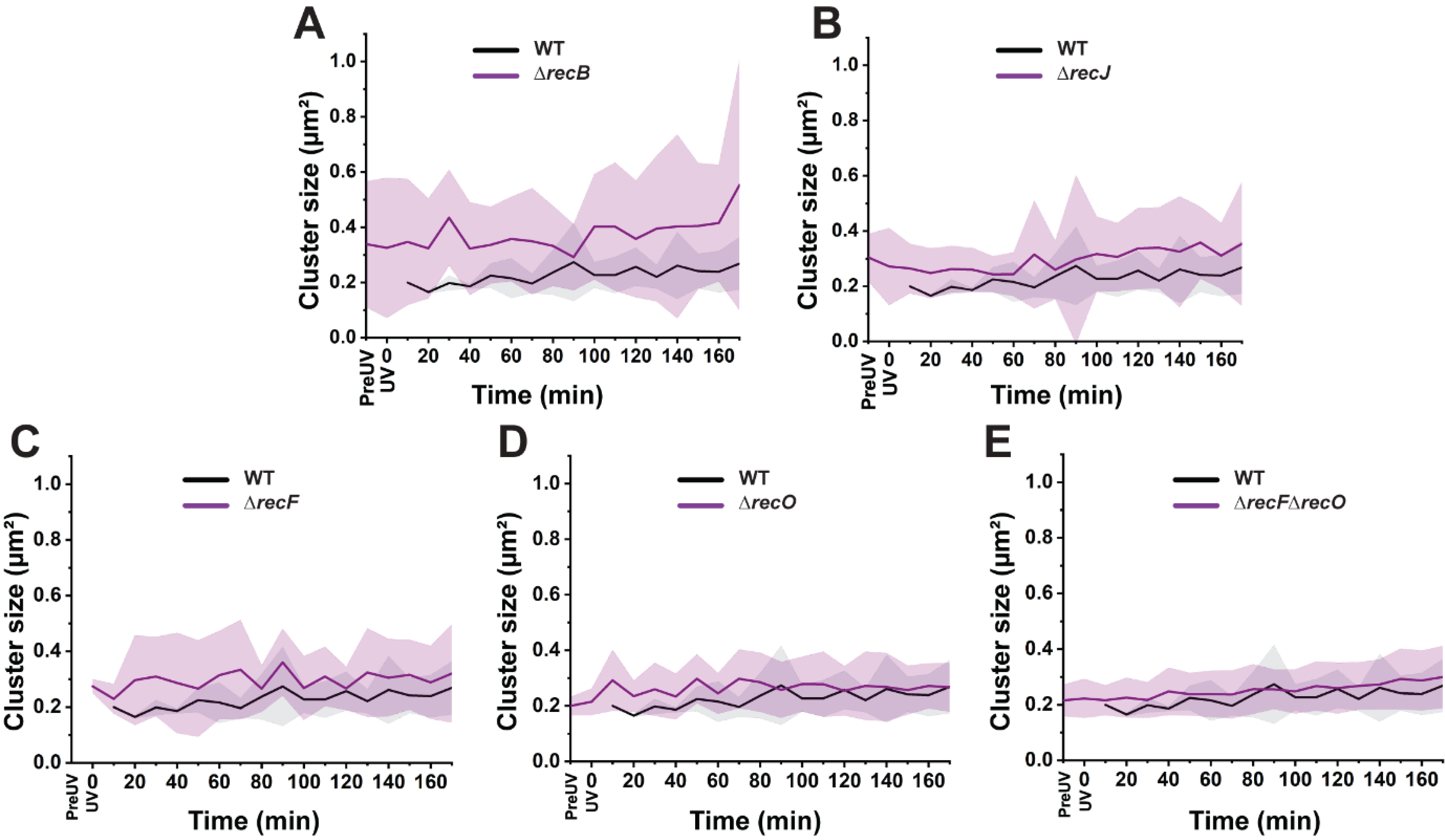

Supplement: S4 Fig — The shaded area represents the standard deviation to emphasize the variation of SSB clusters size within cell population at indicated time points. (TIF) [file pgen.1012109.s004.tif]
