# Supplementary material for: Spatiotemporal characterization of single-stranded DNA Intermediates after UV Irradiation: I: Post-replication gaps formed during slow growth
Source: PLoS Genet. 2026 May 14;22(5):e1012109. doi: 10.1371/journal.pgen.1012109 (PMC13175387; doi:10.1371/journal.pgen.1012109)
Supplement: S1 Movie — The SSB clusters (with an area >16 pixel²) that develop over time are perfectly captured (small yellow outline inside the yellow cell outlines) by thresholding the 16-bit intensity range to 0–6100 arbitrary units (au) and the 8-bit intensity range to 175–255 au for all processed image stacks using Fiji’s default thresholding algorithm. Scale bar represents 2 µm. (PPTX) [file pgen.1012109.s010.pptx]

## Slide 1
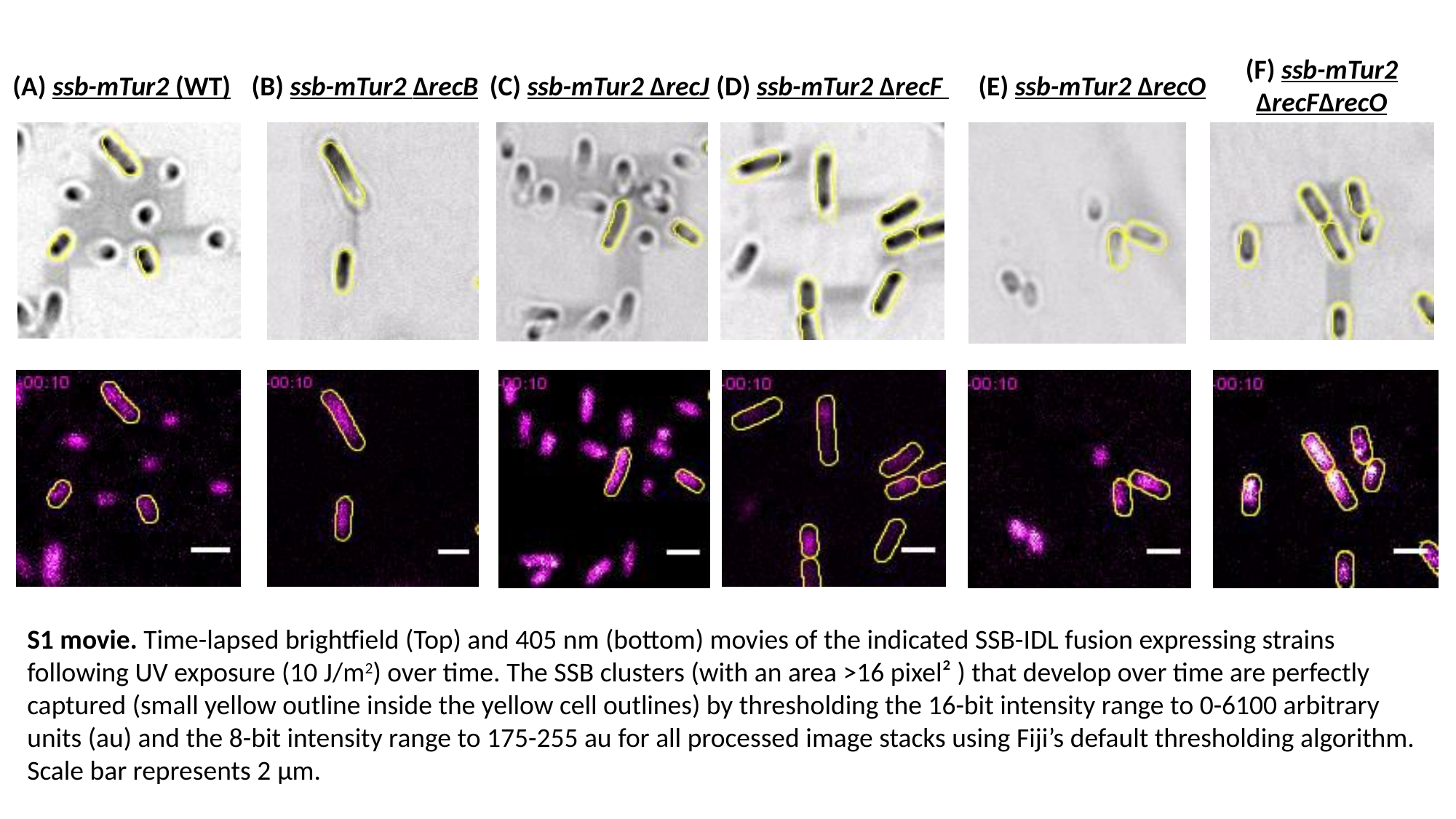

(F) ssb-mTur2 ΔrecFΔrecO
(A) ssb-mTur2 (WT)
(B) ssb-mTur2 ΔrecB
(C) ssb-mTur2 ΔrecJ
(D) ssb-mTur2 ΔrecF
(E) ssb-mTur2 ΔrecO
S1 movie. Time-lapsed brightfield (Top) and 405 nm (bottom) movies of the indicated SSB-IDL fusion expressing strains following UV exposure (10 J/m2) over time. The SSB clusters (with an area >16 pixel² ) that develop over time are perfectly captured (small yellow outline inside the yellow cell outlines) by thresholding the 16-bit intensity range to 0-6100 arbitrary units (au) and the 8-bit intensity range to 175-255 au for all processed image stacks using Fiji’s default thresholding algorithm. Scale bar represents 2 µm.
