# Supplementary material for: Spatiotemporal characterization of single-stranded DNA Intermediates after UV Irradiation: I: Post-replication gaps formed during slow growth
Source: PLoS Genet. 2026 May 14;22(5):e1012109. doi: 10.1371/journal.pgen.1012109 (PMC13175387; doi:10.1371/journal.pgen.1012109)
Supplement: S2 Movie — Images of cells grown in M9 minimal and EZ-rich media at 37 °C were recorded at every 2-minute interval for 28 min whereas, images of cells grown in either M9 minimal or EZ-rich medium at 30 °C were recorded at every 10-minute interval for 170 minutes. All cells images were without exposing to UV. Scale bar represents 2 µm. (PPTX) [file pgen.1012109.s011.pptx]

## Slide 1
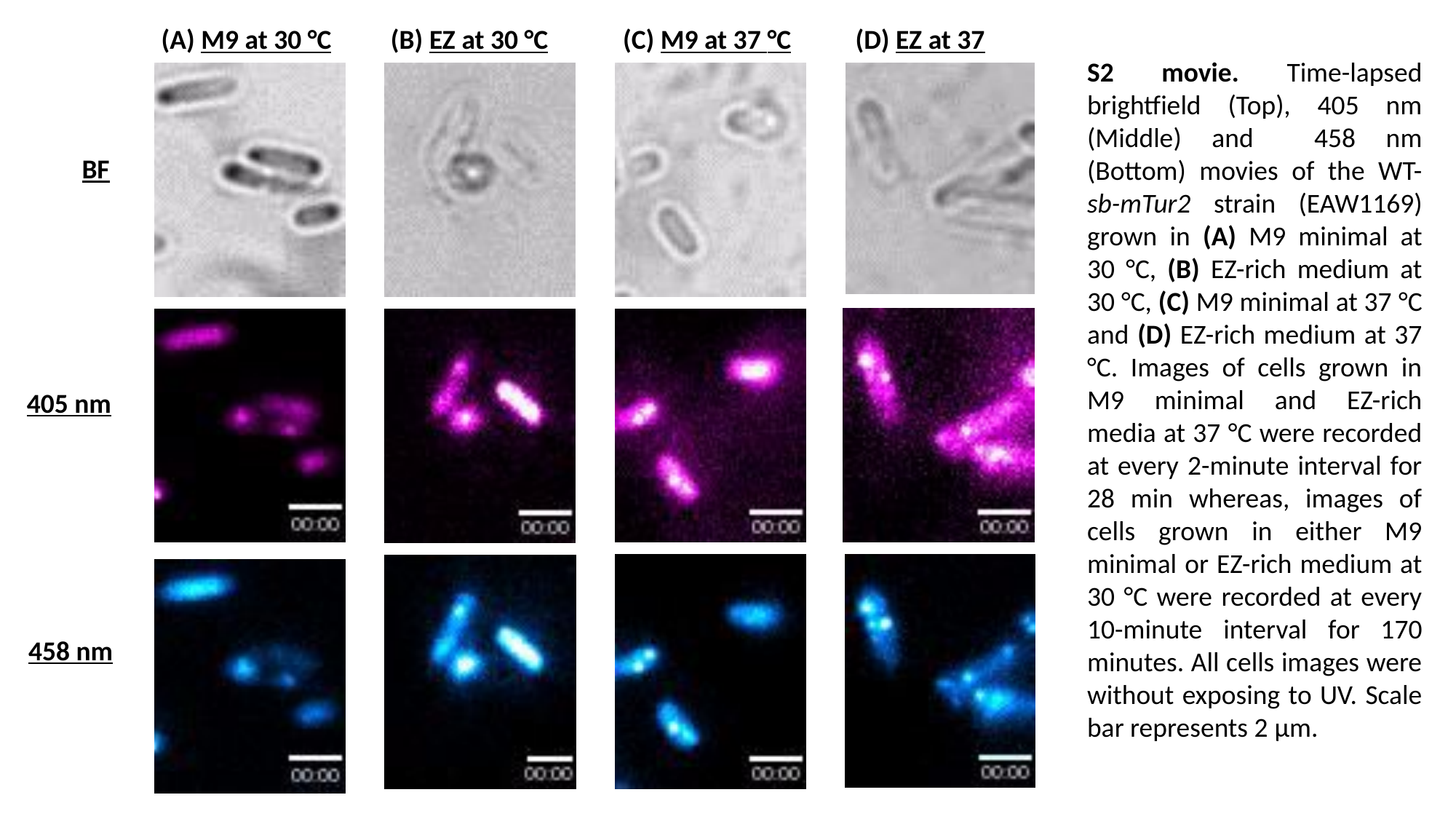

(A) M9 at 30 °C
(B) EZ at 30 °C
(C) M9 at 37 °C
(D) EZ at 37 °C
S2 movie. Time-lapsed brightfield (Top), 405 nm (Middle) and 458 nm (Bottom) movies of the WT-sb-mTur2 strain (EAW1169) grown in (A) M9 minimal at 30 °C, (B) EZ-rich medium at 30 °C, (C) M9 minimal at 37 °C and (D) EZ-rich medium at 37 °C. Images of cells grown in M9 minimal and EZ-rich media at 37 °C were recorded at every 2-minute interval for 28 min whereas, images of cells grown in either M9 minimal or EZ-rich medium at 30 °C were recorded at every 10-minute interval for 170 minutes. All cells images were without exposing to UV. Scale bar represents 2 µm.
BF
405 nm
458 nm
