# Supplementary material for: Spatiotemporal characterization of single-stranded DNA Intermediates after UV Irradiation: I: Post-replication gaps formed during slow growth
Source: PLoS Genet. 2026 May 14;22(5):e1012109. doi: 10.1371/journal.pgen.1012109 (PMC13175387; doi:10.1371/journal.pgen.1012109)
Supplement: S1 Table — (DOCX) [file pgen.1012109.s012.docx]

**S1 Table.** *E.coli* strains used in this study.

| **Strain** | **Relevant Genotype** | **Parent strain** | **Source/technique** |
| --- | --- | --- | --- |
| MG1655 | *recF-wt ssb-wt* (wild type - wt) | - | [1] |
| EAW1169 | *ssb-mTur2::Kan* | MG1655 | [2, 3] |
| EAW1219 | *ssb-mTur2::FRT ∆recB::Kan* | EAW1169 | [2, 3] |
| EAW1463 | *ssb-mTur2::FRT ∆recJ::Kan* | EAW1169 | This study - P1 EAW102 |
| EAW102 | *∆recB::Kan* | MG1655 | [4] |
| EAW820 | *∆recJ::Kan* | MG1655 | [5] |
| CJH0080 | ∆*recF::FRT ssb-mTur2::Kan* | EAW629 | [6] |
| CJH0081 | ∆*recO::FRT ssb-mTur2::Kan* | EAW114 | This study – P1 EAW1169 |
| CJH0082 | ∆*recF::FRT*∆*recO::FRTssb-mTur2::Kan* | EAW668 | This study – P1 EAW1169 |
| EAW668 | ∆*recF::FRT* ∆*recO::Kan* | EAW629 | This study – P1 EAW114 |

All P1 experiments utilized selection for Kanamycin.

References

1. Blattner FR, Plunkett Gr, Bloch CA, Perna NT, Burland V, Riley M, et al. The complete genome sequence of *Escherichia coli* K-12. Science. 1997;277(5331):1453-74.

2. Cherry ME, Dubiel K, Henry C, Wood EA, Revitt-Mills SA, Keck JL, et al. Spatiotemporal Dynamics of Single-stranded DNA Intermediates in *Escherichia coli*. bioRxiv. 2023;Epub:2023/05/22. doi: <https://doi.org/10.1101/2023.05.08.539320> PubMed Central PMCID: PMC37214928.

3. Dubiel K, Henry C, Spenkelink LM, Kozlov AG, Wood EA, Jergic S, et al. Development of a single-stranded DNA-binding protein fluorescent fusion toolbox. Nuc Acids Res. 2020;48(11):6053-67. doi: 10.1093/nar/gkaa320. PubMed PMID: WOS:000574284500024.

4. Henrikus SS, Henry C, McGrath AE, Jergic S, McDonald JP, Hellmich Y, et al. Single-molecule live-cell imaging reveals RecB-dependent function of DNA polymerase IV in double strand break repair. Nuc Acids Res. 2020;48(15):8490-508. doi: 10.1093/nar/gkaa597. PubMed PMID: WOS:000574315100026.

5. Jain K, Wood EA, Cox MM. The *rarA* gene as part of an expanded RecFOR recombination pathway: Negative epistasis and synthetic lethality with *ruvB*, *recG*, and *recQ*. PLoS Genet. 2021;17(12):e1009972. doi: 10.1371/journal.pgen.1009972. PubMed PMID: WOS:000733165400001.

6. Henry C, Kaur G, Cherry ME, Henrikus SS, Bonde NJ, Sharma N, et al. RecF protein targeting to post-replication (daughter strand) gaps II: RecF interaction with replisomes. Nuc Acids Res. 2023;51(11):5714-42. doi: 10.1093/nar/gkad310. PubMed PMID: WOS:000978634400001.
