# Supplementary material for: Spatiotemporal characterization of single-stranded DNA Intermediates after UV Irradiation: I: Post-replication gaps formed during slow growth
Source: PLoS Genet. 2026 May 14;22(5):e1012109. doi: 10.1371/journal.pgen.1012109 (PMC13175387; doi:10.1371/journal.pgen.1012109)
Supplement: S2 Table — (DOCX) [file pgen.1012109.s013.docx]

**S2 Table.** Table list showing the integrated density and SSB copy number of *ssb-mTur2* cells (EAW1169) imaged with 458 nm and 405 nm lasers.
