# Supplementary material for: Spatiotemporal characterization of single-stranded DNA Intermediates after UV Irradiation: I: Post-replication gaps formed during slow growth
Source: PLoS Genet. 2026 May 14;22(5):e1012109. doi: 10.1371/journal.pgen.1012109 (PMC13175387; doi:10.1371/journal.pgen.1012109)
Supplement: S1 Text — (DOCX) [file pgen.1012109.s014.docx]

**Supporting information (S1_Text). Calculations for single-molecule mTur2 integrated intensity at 405 nm to determine SSB cell copy number.**

To calculate the single-molecule mean integrated intensity of SSB, the WT *ssb-mTur2* cell were first imaged under the same conditions previously mentioned by Cherry et al. [1]. Briefly, the cell culture grown in EZ rich medium at 37 °C were loaded onto the APTES-treated flow-cell. Once sufficient cells were adsorbed, fresh nutrient medium was supplemented and the cell images were recorded with 458 nm and 405 nm lasers simultaneously. The first frame of the cell images were background corrected and manual cell outlines were drawn in a MicrobeTracker 0.937 MATLAB plug-in [2] to select in-focus, non-overlapping cells (N= 40 cells). Next, the “‘corrected mean integrated density”’ for each cell (N= 40 cells) imaged at 458 nm was calculated from the signal contained within ROIs representing the outline of the cells. Following this, the corrected mean integrated density of the cells was then divided by the mean single-molecule mTur2 integrated intensity (previously estimated to be 176 au by Cherry et al.) to calculate the estimated number of SSB molecules.

Mean cell integrated density at 458 nm (*I_458_*) = 1556610 ± 692717 arb. Units

Mean cell integrated density at 405 nm (*I_405_*) = 2496498 ± 1101672 arb. Units

Single-molecule mTur2 integrated density of the diffraction limited foci (*I_mTur2-458_)* = 176 arb. Units (95% CI: 66 - 74) [1]

Using equation 1 (*N = I_458_/I_mTur2-458_*), the number of SSB copies (*N*) with cells recorded using 458 nm laser can be calculated as follows

*N = I458/ImTur2-458* = 1556610/176 = 8844 SSBs (~2211 SSB tetramers) per cell

Using equation 2 (*I_mTur2-405_ = I_405_/N*), we can extract the single-molecule mTur2 integrated density of 405 nm cell images.

*I_mTur2-405_ = I_405_/N* = 2496498/8844 = **282 ± 126** **arb. units**

Using the single-molecule mTur2 intergraded density value at 405 nm (282 ± 126 arb. units), we can calculate the estimated SSB copy number within SSB features (foci and clusters) by dividing the mean SSB foci and cluster intensities formed at particular time point with single-molecule mTur2 integrated density value at 405 nm. See Tables A and B for the estimated SSB copy number (in monomers) formed within the SSB foci and clusters of SSB-IDL fusion-expressing strains at the indicated time points.

**Table A**. Estimated SSB copy number (in monomers) within SSB foci of WT and recombination-deficient SSB-IDL fusion strains is provided at indicated time points. SD represents the standard deviation.

**Table B**. Estimated SSB copy number (in monomers) within SSB clusters of WT and recombination-deficient SSB-IDL fusion strains is provided at indicated time points. SD represents the standard deviation.

**Table C.** p-values for cell length differences of Δ*recB*, Δ*recJ*, Δ*recF*, Δ*recO*, and Δ*recFO* relative to WT at the indicated time points.

**Table D.** *p*-values for the percentage change in SSB foci intensities of Δ*recB*, Δ*recJ*, Δ*recF*, Δ*recO*, and Δ*recFO* strains relative to WT at the indicated time points.

**Table E.** *p*-values for the percentage change in SSB cluster intensities of Δ*recB*, Δ*recJ*, Δ*recF*, Δ*recO*, and Δ*recFO* strains relative to WT at the indicated time points.

**Table F.** *p*-values for the percentage change in SSB cluster size of Δ*recB*, Δ*recJ*, Δ*recF*, Δ*recO*, and Δ*recFO* strains relative to WT at the indicated time points.

**Table G.** *p*-values for the percentage change in the number of SSB foci in Δ*recB*, Δ*recJ*, Δ*recF*, Δ*recO*, and Δ*recFO* strains relative to WT at the indicated time points.

**Table H.** *p*-values for the percentage change in the number of SSB clusters in Δ*recB*, Δ*recJ*, Δ*recF*, Δ*recO*, and Δ*recFO* strains relative to WT at the indicated time points.

**Table I.** *p*-values for the percentage change in overall mean foci intensity (top panel), mean cluster intensity (middle panel), and mean cluster size (bottom panel) in Δ*recB*, Δ*recJ*, Δ*recF*, Δ*recO*, and Δ*recFO* strains relative to WT, averaged across the 170-min time course.

1. Cherry, M.E., et al., *Spatiotemporal Dynamics of Single-stranded DNA Intermediates in Escherichia coli.* bioRxiv, 2023. **Epub**: p. 2023/05/22.

2. Sliusarenko, O., et al., *High-throughput, subpixel precision analysis of bacterial morphogenesis and intracellular spatio-temporal dynamics.* Mol Microbiol, 2011. **80**(3): p. 612-27.
